# JAK/BCL2 inhibition acts synergistically with LSD1 inhibitors to selectively target ETP-ALL

**DOI:** 10.1038/s41375-022-01716-9

**Published:** 2022-10-13

**Authors:** Aissa Benyoucef, Katharina Haigh, Andrew Cuddihy, Jody J. Haigh

**Affiliations:** 1 Department of Pharmacology and Therapeutics, Rady Faulty of Health Sciences, University of Manitoba, Winnipeg, MB Canada; 2 CancerCare Manitoba Research Institute, Winnipeg, MB Canada

**Keywords:** Acute lymphocytic leukaemia, Targeted therapies

## Abstract

ETP-ALL (Early T cell Progenitor Acute Lymphoblastic Leukemia) represents a high-risk subtype of T cell acute lymphocytic leukemia (T-ALL). Therapeutically, ETP-ALL patients frequently relapse after conventional chemotherapy highlighting the need for alternative therapeutic approaches. Using our ZEB2^Tg^ ETP-ALL mouse model we previously documented the potential utility of the catalytic LSD1 inhibitor (GSK2879552) for treating mouse/human ETP-ALL. However, this approach proved to be inefficient, especially in killing human LOUCY cell ETP-ALL xenografts in vivo. Here we have revealed the novel involvement of ZEB2/LSD1 complexes in repressing the intrinsic apoptosis pathway by inhibiting the expression of several pro-apoptotic proteins such as BIM (*BCL2L11*) as a major driver for ETP-ALL survival. Treatment with LSD1*i* (particularly with the steric inhibitor SP2509) restored the expression of ZEB2/LSD1 pro-apoptotic BIM (*BCL2L11*) target. In combination with a JAK/STAT pathway inhibitor (JAK*i*, Ruxolitinib) or with a direct inhibitor of the anti-apoptotic BCL2 protein (BCL2*i*, ABT-199) resistance of human and mouse ETP-ALL to LSD1*i* was reversed. This new combination approach efficiently inhibited the growth of human and mouse ETP-ALL cells in vivo by enhancing their differentiation and triggering an apoptotic response. These results set the stage for novel combination therapies to be used in clinical trials to treat ETP-ALL patients.

## Introduction

T-cell Acute Lymphoblastic Leukemia (T-ALL) is an aggressive leukemia accounting for 10–15% of childhood and 25% of adult ALL cases [1]. Previous molecular studies have revealed the presence of four major T-ALL subgroups i.e. the TAL/LMO, the TLX/HOX11, the TLX3/HOX11L2, and the HOXA subgroups [1]. More recently, a new subset of T-ALL named ETP-ALL (Early T cell Progenitor Acute Lymphoblastic Leukemia), that comprises up to 15% of T-ALL, has begun to gain more attention as it represents a high-risk subtype that lacks expression of several T cell surface markers and exhibits aberrant expression of myeloid and stem cell markers. ETP-ALL blasts express CD7, are CD5 dim (<75% positive cells), are absent for CD1a, CD3, CD8, and express one or more myeloid/stem cell related markers (i.e. CD34, CD13 or CD33) [2, 3].

The molecular characterization of ETP-ALL compared to other T-ALL subgroups has revealed distinct alterations, including some gene mutations which are highly enriched in ETP-ALL [4]. In addition, comparison of the gene expression profile of ETP-ALL with normal hematopoietic cells and acute myeloid leukemia demonstrated enrichment for a leukemic stem-cell signature associated with poor outcome in acute myeloid leukemia (AML) [4, 5]. One of the emerging oncogenes that play roles in both AML and ETP-ALL is the transcription factor ZEB2 (Zinc finger E-box-binding homeobox 2) [6]. Although ZEB2 is best known for its role in repressing E-cadherin and promoting epithelial–mesenchymal transition (EMT), recent studies have associated its aberrant expression in AML and ETP-ALL with gross maturational arrest and an aggressive poorly differentiated stem-cell-like leukemia [6, 7]. The role of Zeb2 overexpression in ETP-ALL leukemogenesis was confirmed by using Cre/loxP-based conditional approaches to overexpress Zeb2 from the Rosa26 locus [6] in the entire hematopoietic system or solely in T-cells [6, 8]. The leukemic cells obtained from these mice are characterized by an early block in T-cell development [8] and have a cell surface antigen repertoire and gene expression profile that closely resembles those seen in ETP-ALL patients [6].

From a clinical perspective ETP-ALL is associated with a significantly worse outcome in children and young adults compared with other T-ALL subtypes [4, 9, 10]. Clearly these outcomes highlight the need for alternative therapeutic approaches to improve the treatment of this high-risk, poor prognosis group. Epigenetic drugs (with potent anticancer activity) have emerged as a novel therapeutic approach for cancers with poor prognosis. Previously, we unveiled an interaction of ZEB2 with the NuRD epigenetic complex and its enzymatic subunits (e.g. LSD1/KDM1A, HDAC1/2) that is known to be mainly implicated in gene repression [11]. Surprisingly, although a transgenic mouse ETP-ALL cell line that overexpresses Zeb2 (Zeb2^Tg^), as well as human derived ETP-ALL cell lines (e.g. LOUCY) that have increased ZEB2 expression, are relatively sensitive in vitro to a catalytic LSD1 inhibitor, human ETP-ALL LOUCY cells were unresponsive to this drug in vivo [11].

Given the critical role of the tumor microenvironment (TME) in resistance to therapy in vivo, we hypothesized that extrinsic factors might be involved in the resistance mechanism of ETP-ALL to LSD1 inhibitors (LSD1*i*). We demonstrated that the IL7/IL7R signaling pathway plays a role in enabling ETP-ALL cells to resist LSD1*i*-induced cell death. In addition, we identified molecular crosstalk between IL7/IL7R signaling and the ZEB2/LSD1/NuRD complex in the regulation of the intrinsic apoptosis pathway via the JAK/STAT pathway. Interestingly, global assessment of H3K4 methylation associated with LSD1 showed an aberrant increase in euchromatic H3K4me2 compared to active H3K4me3 marks in both human and mouse ETP-ALL cells which suggests a unique repression mechanism of ‘poised” genes previously observed in hematopoietic progenitors [12, 13], that could be appropriated by the ZEB/LSD1 complex to repress the expression of certain key genes. One of the key targets that ZEB2/LSD1 directly represses is the pro-apoptotic *BCL2L11* (BIM) gene, and consequently confers a selective sensitivity of ETP-ALL to LSD1*i* in vivo. Conversely, targeting upstream components of the JAK/STAT pathway or directly interfering with the pro-survival BCL2 protein with small molecule inhibitors sensitizes mouse and human ETP-ALL to LSD1*i-*induced cell death both in vitro and in vivo. These results suggest that ZEB2 levels are responsible for directly (BIM) and indirectly (BCL2) maintaining pro-survival programs in ETP-ALL settings. In addition, we unveiled for the first time the importance of a scaffolding role of LSD1 in human ETP-ALL that has been previously reported in other cancer settings [14, 15].

## Results

### Defining the involvement of extrinsic factors in resistance of ETP-ALL to LSD1 inhibitors (LSD1*i*)

We previously reported that the ZEB2 oncogene interacts with the NuRD complex and its non-core LSD1 subunit in mouse ETP-ALL induced by Zeb2^Tg^ [11] which was further confirmed using immunoprecipitation (IP) of ZEB2 with LSD1 in our derived Zeb2^Tg^ ETP-ALL cell lines (Fig. S1A). Reducing the expression of ZEB2 via shRNA-based knock-down specifically inhibited the growth of Zeb2^Tg^ ETP-ALL after 10 days of culture in complete medium (Fig. S1B–D). These findings indicated a potential therapeutic strategy of targeting ZEB2 transcriptional complexes for irradicating ETP-ALL leukemia. LSD1 (KDM1A) has been found to be overexpressed in several hematological and solid tumors and shown to be required for maintenance of leukemia (e.g. AML) [16]. Several inhibitors of LSD1 (LSD1*i*), designed to either target its catalytic or scaffolding activity, have been developed and are currently used in clinical trials [14].

**Fig. 1:**
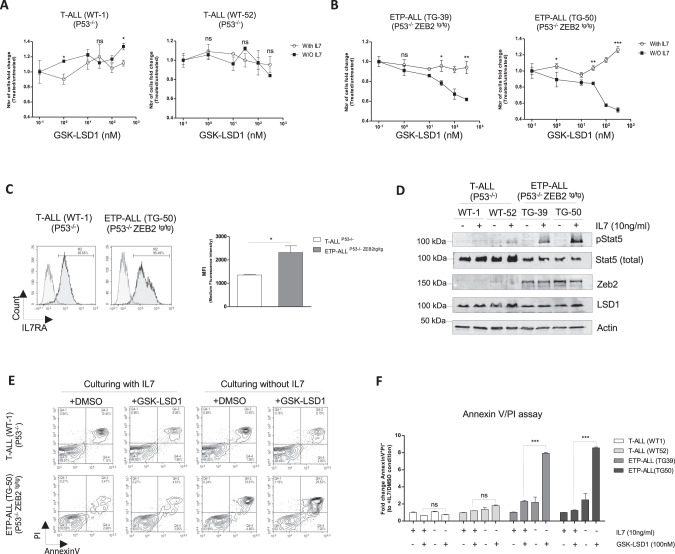
IL7/IL7R signaling drives the resistance of ZEB2Tg ETP-ALL cells to GSK-LSD1 (LSD1 demethylase inhibitor). Viability assays in the T-ALL (P53^−/−^) (**A**) and ETP-ALL (ZEB2^tg/tg^ P53^−/−^) cell lines (**B**) treated for 48 h with (open circles) or without (black squares) IL7 cytokine (10 ng/ml) plus increasing concentrations of GSK-LSD1. **C** Left-Flow cytometry-based measurement of cell surface IL7R expression in T-ALL (P53^−/−^) and ETP-ALL (ZEB2^tg/tg^ P53^−/−^) cell lines, right-average expression from 3 separate analyses (**D**) Western blot analysis of T-ALL (P53 and ETP-ALL (ZEB2^tg/tg^ P53^−/−^) cell lines stimulated with IL-7 detecting phosphorylated and total STAT5, ZEB2 and LSD1. **E** AnnexinV/PI assay measuring apoptosis in T-ALL (P53^−/−^) and ETP-ALL (ZEB2^tg/tg^ P53^−/−^) cell lines treated with GSK-LSD1 with or without IL7 cytokine for 48 h. **F** Summary panel of normalized apoptosis measurement from 3 independent experiments. *p < 0.05, **p < 0.01, ***p < 0.001.

Taking advantage of such inhibitors, we previously showed that treatment of Zeb2^High^ ETP-ALL cells with the catalytic LSD1*i* GSK2879552 (hereafter named GSK-LSD1) induces a highly variable pattern of response. Indeed, GSK-LSD1 affected the cell growth of Zeb2^Tg^ ETP-ALL only after 7 days of culture and did not induce any significant effects on the human ETP-ALL cell line LOUCY in vivo [11]. We presumed that this latency in response in vitro was related to the deprivation of culture medium from its cytokines (mainly IL7) or nutrients during this long period of treatment. In addition, the IL7 cytokine is an essential factor for leukemic cell growth that might be highly consumed during cultures as also seen in vivo upon leukemia development [17, 18], which lead us to the hypothesis that this extrinsic factor may play an important role in the resistance process of Zeb2^Tg^ ETP-ALL to LSD1*i*. To address this question, we cultured Zeb2^Tg^ ETP-ALL or mature T-ALL cells (as specificity controls) with increasing concentrations of the GSK-LSD1*i*, in the presence or absence of the IL7 cytokine. In accordance with our hypothesis, we observed that in the absence of IL7, GSK-LSD1*i* impacted the growth of Zeb2^Tg^ ETP-ALL within 48 hours and this effect was enhanced at high concentrations (Fig. 1A, B). These results were confirmed with the two other LSD1*i* (ORY1001 and SP2509) (Fig. S1E, F). In addition, we confirmed that the increased expression and activity of IL7/IL7R signaling [11] in Zeb2^Tg^ ETP-ALL compared to mature T-ALL (Fig. 1C, D) indicating that ETP-ALL are indeed particularly sensitive to IL7. By using Annexin V/PI analysis, we also confirmed that the lack of IL7/IL7R signaling dramatically synergizes the anti-leukemic activity of LSD1*i* in Zeb2^Tg^ ETP-ALL by inducing apoptosis (Fig. 1E, F).

### Deciphering the interplay of IL7/IL7R signaling, ZEB2 and LSD1 inhibition in driving apoptosis in ETP-ALL apoptotic responses

We first interrogated existing Chromatin immunoprecipitation (ChIP) data for ZEB2, LSD1 and STAT5b (downstream effector of IL7/IL7R signaling) that are publicly available from the ENCODE3/4 projects [19]. We observed that over 35% (*P* value < 0.01) of ZEB2-binding sites are co-occupied by LSD1 whereas only ~0.15% are co-occupied by STAT5b (Fig. S2A, C). Furthermore, gene ontology analysis with GREAT (Genomic Regions Enrichment of Annotations Tool) [20] of the common genes regulated by ZEB2/LSD1/STAT5b (associated with 21178 common peaks) (Table S1) revealed their involvement in the regulation of intrinsic apoptotic signaling pathways (Binomial Raw *P* Value = 8.94E^−45^), cytoplasmic (Binomial Raw *P* Value = 2.66E^−34^) and mitochondrial (Binomial Raw *P* Value = 1.17E^−33^) membrane permeability (Fig. S2D, E, Table S1).

**Fig. 2:**
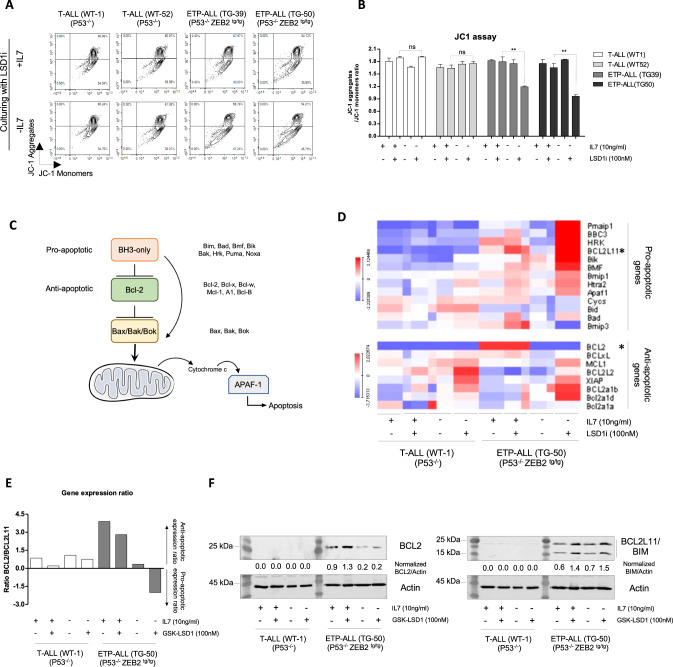
Synergetic effects of IL7 withdrawal with GSK-LSD1 to specifically induce ETP-ALL cell apoptosis. **A** Analysis of mitochondrial membrane potential by JC-1 staining using flow cytometry; (**B**) Statistical analysis of flow cytometry data of JC-1 staining stability and reduction of JC-1 aggregates/JC-1 monomers that correlates with defects in mitochondrial membrane (**) *P* < 0.01. **C** Illustrative schema of molecular interplay between the pro- (BH3-only proteins) and anti-apoptotic proteins (Bcl2 protein) to interact with effectors proteins (Bax/Bak/Bok) that disturbs the mitochondrial membrane, triggering the intrinsic apoptosis pathway. **D** Gene expression heat map of proteins involved in the intrinsic apoptosis pathway by RT-qPCR in T-ALL (P53^−/−^-WT-1) and ETP-ALL (ZEB2^tg/tg^ P53^−/−^-TG-50) cell lines cultured with/without IL7 cytokine (10 ng/ml) in combination with GSK-LSD1 (100 nM). **E** the gene expression ratio of anti-apoptotic BCL2 protein and pro-apoptotic BIM (*Bcl2l11*) protein measured by RT-qPCR. **F** Western blot assessment of expression of anti-apoptotic BCL2 protein and pro-apoptotic BIM (*Bcl2l11*) protein in T-ALL (P53^−/−^-WT-1) and ETP-ALL (ZEB2^tg/tg^ P53^−/−^-TG-50) cell line cultured with/without IL7 cytokine (10 ng/ml) in combination with GSK-LSD1 (100 nM).

To confirm that the apoptosis induced by LSD1*i* and the lack of IL7 stimulation in Zeb2^Tg^ ETP ALL is triggered by the intrinsic (mitochondrial dependent) pathway, we used JC-1 cell-permeable staining to assess mitochondrial membrane potential. The results confirmed that the apoptosis induced by LSD1*i* (GSK-LSD1*i*) in absence of IL7/IL7R signaling is associated with mitochondrial membrane damage (Fig. 2A, B). In addition, we applied qPCR analysis to assess the dynamic expression of ~21 genes involved in intrinsic/mitochondrial apoptosis pathway (Fig. 2C).

Interestingly, we noticed strong expression of the anti-apoptotic *BCL2* gene in the presence of IL7 in Zeb2^Tg^ ETP-ALL cells; in contrast, treatment with LSD1*i* activated the transcription of several genes encoding for pro-apoptotic proteins (e.g. *Bcl2l11*/BIM) (Fig. 2D, E), findings that were confirmed at the protein level (Fig. 2F). To determine the relevance of these findings in the human ETP-ALL context we compared the expression of BCL2, BCL2L1 (BCL-XL), MCL1 pro-survival factors and BCL2L11 (BIM) pro-apoptotic factor in ETP-ALL (*N* = 12) and mature T-ALL (*N* = 40) patient samples (Fig. S3A). BCL2 is significantly higher in ETP-ALL samples compared to mature T-ALL (*p* < 0.0001) whereas BCL2L1 (BCL-XL) levels are the same and MCL1 is significantly although marginally higher in ETP-ALL settings versus mature T-ALL (*p* < 0.0135). The pro-apoptotic BCL2L11 (BIM) is significantly lower in expression in ETP-ALL samples compared to mature T-ALL (*p* < 0.0015).

IL-7 stimulation was also demonstrated to specifically stimulate *Bcl2* (19.7-fold) and *Bcl2l1* (2-fold) expression in Zeb2^Tg^ ETP-ALL cells but not mature T-ALL. *Mcl1* and *Bcl2l11* levels did not change in either cell population following IL-7 stimulation (Fig. S3B).

### Exploring the therapeutic potential of targeting JAK/STAT pathway (IL7/IL7R signaling) with small molecular inhibitors to sensitize Zeb2^Tg^ ETP-ALL to LSD1*i* (GSK-LSD1)

To test the therapeutic potential of this mechanism, we assessed whether JAK/STAT pathway inhibition sensitizes Zeb2^Tg^ ETP-ALL to LSD1*i*. Several drugs were tested in vitro for their specificity, toxicity, and for their synergy with LSD1*i* to specifically compromise the growth and viability of Zeb2^Tg^ ETP-ALL. Ruxolitinib (FDA approved, known as a dual inhibitor of JAK1/JAK2, named hereafter JAK*i*) [21] is one of the most promising drugs as it efficiently reduced the activation of IL7/IL7R signaling through a significant reduction of STAT5 phosphorylation (Fig. S3C) and STAT5 target gene expression (e.g. BCL2) in Zeb2^Tg^ ETP-ALL (Fig. S3C). Use of JAKi was as effective in blocking as using an anti-IL-7R antibody in blocking pSTAT5 and BCL2 expression in Zeb2^Tg^ ETP-ALL cells (Fig. S3C). JAKi and anti-IL-7R antibody had no effects on steady state BCL2L1 expression in mature T-ALL or ETP-ALL cells but did decrease MCL1 expression specifically in Zeb2^Tg^ ETP-ALL cells (Fig. S3C).

**Fig. 3:**
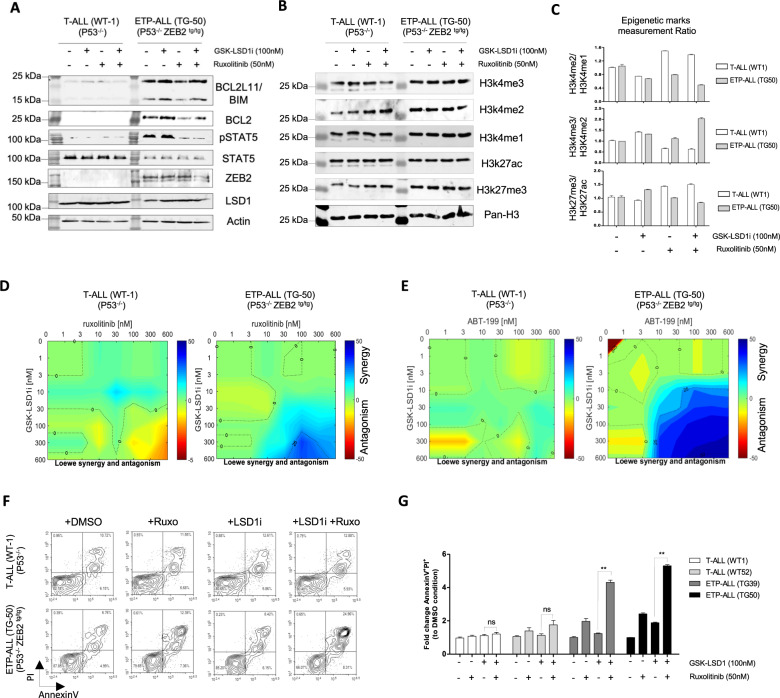
Exploring the therapeutic potential of GSK-LSD1/Ruxolitinib and GSK-LSD1/ABT-199 drug combinations to selectively kill Zeb2Tg ETP-ALL cells. Assessing the anti-apoptotic BCL2, pro-apoptotic BIM (*Bcl2l11*), phosphoSTAT5 (nuclear), total STAT5, ZEB2 and LSD1 proteins (**A**) and Epigenetic marks (**B**) in T-ALL (P53^−/−^-WT-1) and ETP-ALL (ZEB2^tg/tg^ P53^−/−^-TG-50) cells cultured with Ruxolitinib (50 nM) in with/without GDK-LSD1 (100 nM). **C** Quantification of the epigenetic marks illustrated by the ratios H3K4me2:H3K4me1, H3K4me3:H3K4me2 and H3K27me3:H3K27ac to assess the dynamics of methylation and acetylation of histones in T-ALL (P53^−/−^-WT-1) and ETP-ALL. Cells were treated with the indicated concentrations Ruxolitinib (**D**) or ABT-199 (**E**) (concentration above the x-axis, GSK-LSD1 concentration along the y-axis) and assessed with trypan blue dye. Cell growth data were normalized to untreated control wells on each plate. Synergy was analyzed using Combenefit software with Loewe model *n* = 3. **F** Annexin V/PI assay measuring apoptosis in T-ALL (P53^−/−^) cell lines and ETP-ALL (ZEB2^tg/tg^ P53^−/−^) cell lines, and (**G**) Summary panel of normalized measurement of AnnexinV/PI^+^ cells in each cell line treated with Ruxolitinib (50 nM) with/without GSK-LSD1 (100 nM). *N* = 3, **p < 0.01.

The combination of Ruxolitinib and GSK-LSD1 was further tested for its efficiency to reverse the expression of anti- and pro-apoptotic proteins (BCL2 & BIM) in Zeb2^Tg^ ETP-ALL. Accordingly, the treatment with Ruxolitinib (JAK*i*) reduced efficiently the expression of pro-survival BCL2 protein to a similar extent as IL7 withdrawal, and the combination with GSK-LSD1 totally reversed the protein ratio BCL2/BIM in Zeb2^Tg^ ETP-ALL cells (Fig. 3A). Moreover, LSD1 inhibition with GSK-LSD1 reduced globally the H3K4me2 mark in Zeb2^Tg^ ETP-ALL, and in combination with Ruxolitinib (JAK*i*) reduced slightly the repressive mark H3K27me3 but without major changes in the active marks H3K27ac and H3K4me3/1. All these observed changes were opposite to the effects of these drugs seen in mature T-ALL (Fig. 3B, C, Fig. S5C, D).

Furthermore, treatment with increasing concentrations of Ruxolitinib (0 to 600 nM) and LSD1*i* (GSK-LSD1, 0–600 nM) confirmed their high synergy to reduce cell growth (Fig. 3D) and to specifically induce apoptosis in Zeb2^Tg^ ETP-ALL (Fig. 3F, G). Taking advantage of ABT-199 (also known as Venetoclax) [22], an available drug designed to target specifically the BH3-domain of BCL2, we confirmed that the drug resistance of Zeb2^Tg^ ETP-ALL to LSD1*i* is active essentially through BCL2 protein that is regulated by IL7/IL7R through JAK/STAT pathway (Fig. 3E). We next examined the effects on cell growth (Fig. S4A) and cell death (Fig. S4B, C) of murine T-ALL (WT-1) and ETP-ALL (Tg-50) cells after treatment with increasing concentrations of a specific inhibitor of MCL1 (AZD5991), BCL2L1 (WEHI-539), in comparison with BCL2 inhibition (ABT-199) in the presence or absence of GSK-LSD1 inhibitor (100 nm). As expected, the various drug combinations had little effects on cell growth (Fig. S4A, top panel), or survival of mature T-ALL (Fig. S4B). The drug combination ABT-199/GSK-LSD1 that inhibits BCL2 and LSD1 had the greatest effects on decreasing cell growth (Fig. S4, lower panel) and increasing apoptosis (Fig. S4C) compared to simultaneous inhibition of LSD1 and either MCL1 or BCL2L1.

### The role of ZEB2/LSD1 and IL7/IL7R signaling in the regulation of the intrinsic apoptosis pathway in ETP-ALL

To further confirm the molecular crosstalk between JAK/STAT pathway (IL7/IL7R signaling) and ZEB2/LSD1 in regulating the intrinsic apoptosis pathway in ETP-ALL, we used the ENCODE publicly available ChIP-seq data (Fig. S5A). The computational analysis of this data revealed that LSD1 genomic binding overlapped with ZEB2 at the *BCL2L11* (BIM) locus. The BIM pro-apoptotic factor has been shown to play a critical role in glucocorticoid-induced apoptosis in normal and malignant lymphoid cells through its antagonistic role to the anti-apoptotic BCL2 protein that is increased by IL7/IL7R signaling in ETP-ALL [23]. Furthermore, we also noticed the binding of STAT5b (downstream IL7/IL7R signaling) to enhancer regions associated with the *Bcl2* locus (Fig. S5A).

**Fig. 4:**
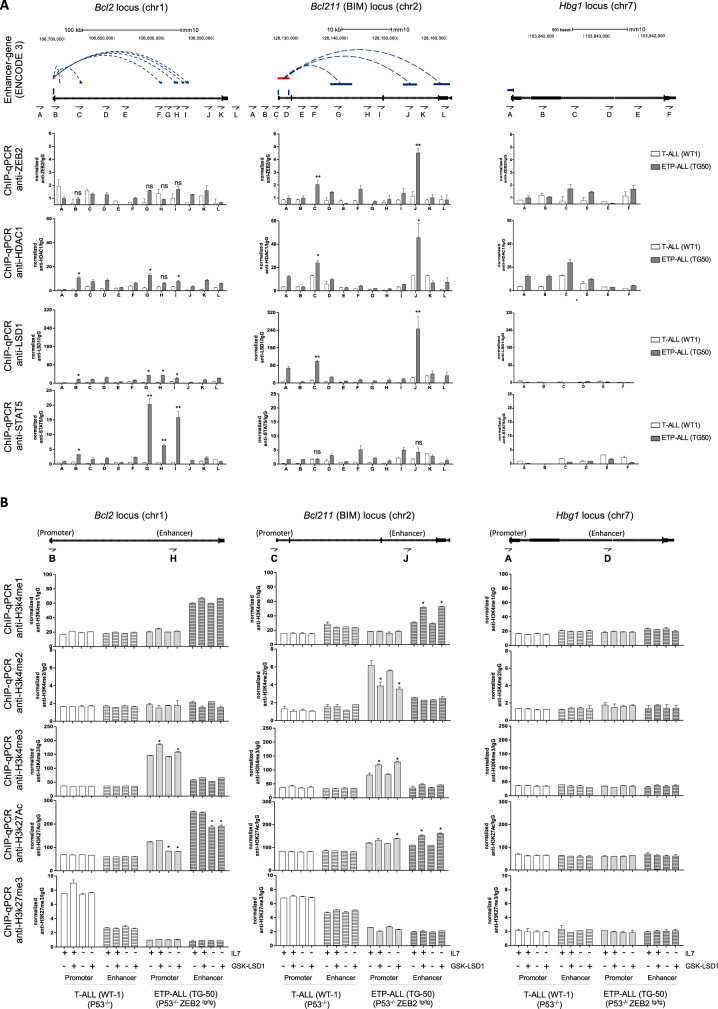
Assessment of ChIP peaks and methylation marks in control mature T-ALL and Zeb2Tg immature ETP-ALL cells. **A** ChIP-qPCR of ZEB2, LSD1 and STAT5b in anti-apoptotic *Bcl2*, pro-apoptotic *Bcl2l11(BIM)* and control gene *Hgb1* locus to assess their binding in ENCODE Candidate Cis-Regulatory Elements (cCREs) combined from all cell types (based on ENCODE data^19^). **B** Assessing the dynamics of epigenetic marks in highlighted regions (Promoter and enhancer for each locus) in T-ALL (P53^−/−^-WT-1) and ETP-ALL (ZEB2^tg/tg^ P53^−/−^ -TG50) cell lines cultured with/without IL7 cytokine (10 ng/ml) in combination with GDK-LSD1 (100 nM) treatment. *p < 0.05, **p < 0.01.

To further validate these observations in Zeb2^Tg^ ETP-ALL cells, we first examined ZEB2, LSD1, HDAC1 and STAT5b binding using the ENCODE Candidate Cis-Regulatory Elements (cCREs) [24] associated with the *Bcl2*, *Bcl2l11* (BIM) and *Hbg1* loci (the latter being a control gene) by using ChIP-qPCR (Fig. S5B). The obtained results confirmed the binding of STAT5b at the enhancer regions (*Bcl2* + 100Kb, +105Kb, +110Kb herein named region “G” “H” “I” respectively) associated to *Bcl2* locus. However, ZEB2 with its co-factors LSD1 and HDAC1, both being enzymatic subunits of the NuRD complex, are more enriched in promoter and enhancer regions (*Bcl2l11* + 26Kb herein named region “J”) associated with the *Bcl2l11* (BIM) locus (Fig. 4A).

Furthermore, the assessment of epigenetic marks in these regions reveled an enrichment of the repressive mark H3K27me3 at the promoter of *Bcl2* gene in mature T-ALL, whereas enrichment of active H3K27ac and H3K4m3 marks were more prominent in the Zeb2^Tg^ ETP-ALL.

Interestingly, in the absence of IL7/IL7R signaling there were specific reductions in the active H3K27ac mark at the enhancer (*Bcl2* + 105Kb herein named region “H”) bound by STAT5b, which correlates with decreased expression of the anti-apoptotic *Bcl2* gene. The promoter of *Bcl2l11* (BIM) gene also showed an enrichment of the repressive mark H3K27me3 in mature T-ALL whereas in the Zeb2^Tg^ ETP-ALL, we noticed more an enrichment of H3K4me2 mark and reduction in the active H3K4me3 mark (Fig. S5C, D), which is known to be the H3K4 methylation pattern in TSS (promoter) regions of “poised” genes [13, 25]. Interestingly, the treatment with GSK-LSD1 released this repression by increasing the active marks H3K4me3/1 at the promoter and enhancer regions (*Bcl2l11* + 26Kb) bound by ZEB2 and its co-factors (LSD1 and HDAC1), which correlated with an increase in expression of pro-apoptotic BIM protein (Fig. 4B).

Collectively, these data led to a model proposing that the hyper-activation of IL7/IL7R signaling (JAK/STAT pathway) can increase the expression of the anti-apoptotic BCL2 protein through the accumulation of H3K27ac active marks at an enhancer (e.g. *Bcl2* + 105Kb) bound by STAT5b, and this can play an antagonistic role to LSD1*i* that reactivates the expression of pro-apoptotic genes, which are normally repressed by the ZEB2/LSD1/NuRD complex in the context of Zeb2^Tg^ ETP-ALL. This occurs through the prevention of the accumulation of the H3K4me3/1 active marks at the promoters and enhancers of its target genes such as *Bcl2l11* (BIM).

### Pre-clinical validation of combinatorial drugs to target Zeb2^Tg^ ETP-ALL in vivo

**Fig. 5:**
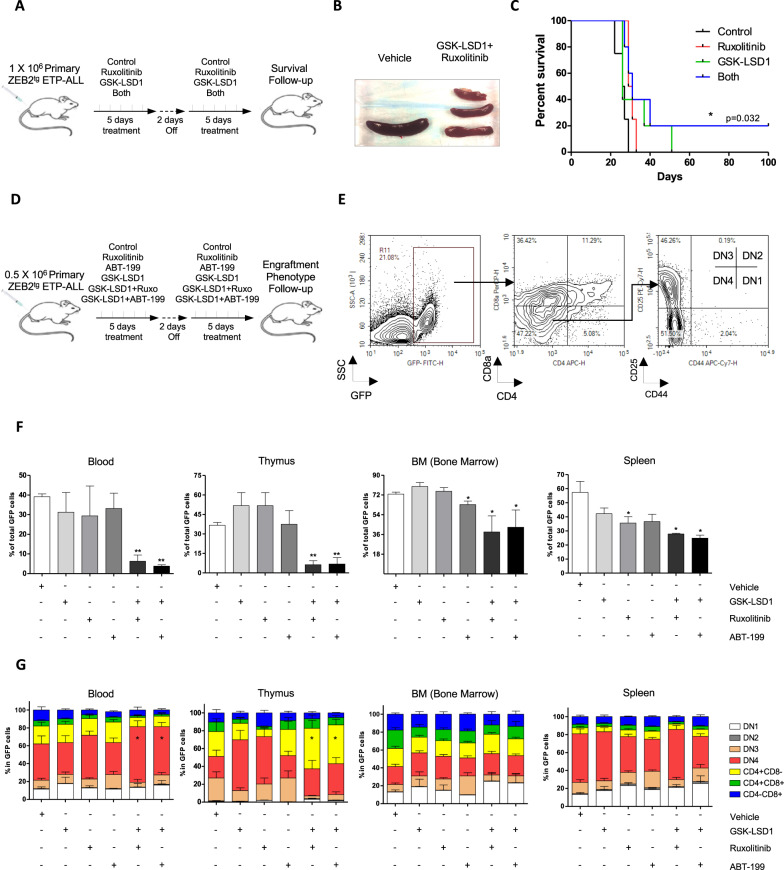
Drug synergy of the drug combination LSD1i (GSK-LSD1) with JAKi (Ruxolitinib) or BCL2i (ABT-199) in vivo. **A** Experimental strategy. C57BL/6 J mice were transplanted by intravenous (IV) injection of 1.0 × 10^6^ primary C57BL/6 J ZEB2^tg^ ETP-ALL tumour cells (cKIT^+^eGFP^+^cells). The recipient mice were treated by oral gavage with vehicle control, GSK-LSD1 (1.5 mg/kg/day) and/or Ruxolitinib (30 mg/kg/ twice a day) for 2 consecutive weeks. **B** the drug combination reduced efficiently the splenomegaly and (**C**) improved overall survival *n* = 5 mice/condition, *p < 0.032. **D** Experimental strategy. Recipient mice engrafted with 0.5 × 10^6^ of primary C57BL/6 J ZEB2^tg^ ETP-ALL cells (cKIT^+^eGFP^+^cells^)^, were treated by gavage with vehicle control, GSK-LSD1 (0.5 mg/kg/day) and/or Ruxolitinib (30 mg/kg/ once a day), ABT-199 (25 mg/kg/ once a day) for 2 consecutive weeks. *n* = 5 mice/condition (**E**) Gating strategy of primary C57BL/6 J ZEB2^tg^ ETP-ALL tumour cells analysed by flow cytometry. **F** Assessing the engraftment of primary C57BL/6 J ZEB2^tg^ ETP-ALL/GFP^+^ cells in mice treated with single or combination of drug and (**G**) their impact on the differentiation of primary ZEB2^tg^ ETP-ALL/GFP^+^ cells in vivo*.* *p < 0.05, **p < 0.01.

As the in vitro data showed that Ruxolitinib/JAK*i* can synergize with LSD1*i* to induce efficient apoptosis of Zeb2^Tg^ ETP-ALL, we next tested whether this holds also true in vivo. First, we assessed whether the combination of GSK-LSD1*i* and Ruxolitinib or ABT-199 for two weeks produced any hematologic abnormalities or overt toxicity in healthy mice, which appeared to not be the case (Fig. S6A, C). We next investigated the efficacy of this GSK-LSD1*i* (1.5 mg/kg/day) [26] and/or Ruxolitinib (30 mg/kg/ twice a day) [27] drug combination in syngeneic C57BL/6 J wild-type (WT) mice transplanted with primary C57BL/6 J ZEB2^Tg^ ETP-ALL tumour cells for 2 consecutive weeks (Fig. 5A). Combining GSK-LSD1 with Ruxolitinib considerably reduced the splenomegaly observed in this aggressive disease model (Fig. 5B), and enhanced mouse survival compared to either drug alone (Fig. 5C, *p = 0.032).

To further understand the impact of this combination therapy on the early DN3/4 T cell differentiation block observed in ZEB2^Tg^ ETP-ALL model [8], we analysed the phenotype of ZEB2^Tg^ ETP-ALL tumour cells isolated from blood, thymus, bone marrow (BM) and spleen of treated and non-treated recipient mice (Fig. 5D, E). Interestingly, the combination therapy (GSK-LSD1*i* with Ruxolitinib or ABT-199) efficiently reduced the levels of leukemic (GFP^+^) cells in all tested tissues compared to single drug treatments (Fig. 5F). Furthermore, enhanced differentiation of ZEB2^Tg^ ETP-ALL was detected upon GSK-LSD1*i* with Ruxolitinib or ABT-199 in the blood (higher levels of DN4 T cells) and in the thymus (enhanced SP CD4^+^CD8^−^ levels). However, these changes were not observed in the BM and spleen (Fig. 5G), suggesting that other tissue-specific factors may interfere with the differentiation of treated ZEB2^Tg^ ETP-ALL tumour cells in vivo.

### Scaffolding role of LSD1 in human ETP-ALL leukemia

To test our therapeutic strategy in human ETP-ALL contexts, we used LOUCY cells that are a unique human derived ETP-ALL cell line, and the well characterized mature T-ALL JURKAT and/or MOLT3 as control cell lines. The LOUCY cell line has high levels of ZEB2 as well as other ETP-ALL specific transcription factors such as LYL1, LMO2, and HHEX [6], that is like primary ETP-ALL patient samples in comparison to mature T-ALL (Fig. S7A). Like Zeb2^Tg^ mouse ETP-ALL cell lines, knock-down of ZEB2 in LOUCY cells dramatically decreased cellular expansion over a 12-day period (Fig. S7 B, E).

**Fig. 6:**
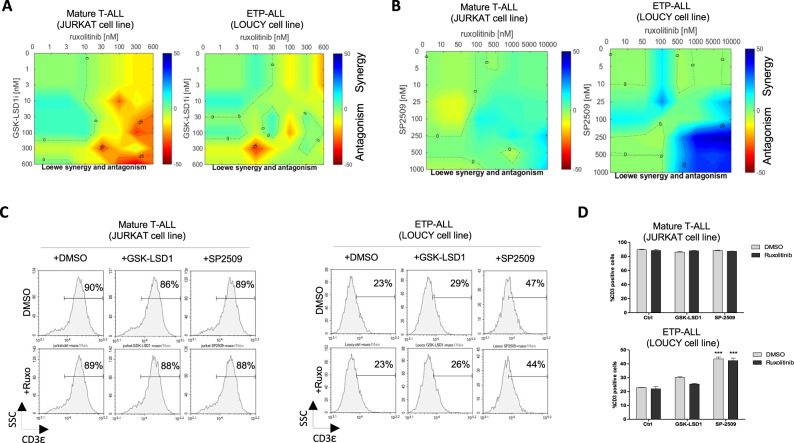
Drug synergy experiments in human mature JURKAT T-ALL and LOUCY ETP-ALL cells. Cells were treated with the indicated concentrations (Ruxolitinib-JAK*i*) concentration above the x-axis, GSK-LSD1 or SP2509 concentration along the y-axis) and cell growth data were normalized to untreated control wells on each plate. **A** Drug synergy analysis was performed using Combenefit software confirmed total absence synergy of enzymatic LSD1 inhibitor (GSK-LSD1) with JAK*i*. In contrast to the scaffolding LSD1 inhibitor (SP2509) where high synergy was demonstrated in human LOUCY ETP-ALL (**B**). **C** Flow cytometry measurement of the efficiency of these two combinatorial drugs (LSD1 inhibitors) to induce the differentiation of human ETP-ALL in vitro by assessing the CD3ε differentiation marker of T-cells, (**D**) Efficiency of scaffolding LSD1 inhibitor (SP2509) to induce the differentiation human LOUCY cell line compared to catalytic LSD1 inhibitor (GSK-LSD1) in combination or not with JAK*i*, and as control we used the mature Jurkat T-ALL*. n* = 3 (***) *P* < 0.001.

We found that the combination of catalytic LSD1 inhibitor (GSK-LSD1*i*, 0–600 nM) with Ruxolitinib (0–600 nM) was not effective for the in vitro synergistic killing of the human LOUCY ETP-ALL cells (Fig. 6A) within a 72-h period, and this held true at high drug concentrations (Fig. S8A) or using ORY-1001 another catalytic LSD1 inhibitor (Fig. S8B). We have previously demonstrated that extended exposure (up to 24 days) of LOUCY cells to this GSK-LSD1 inhibitor is required to elucidate effective an effective killing response [11].

However, using the noncovalent scaffolding LSD1 inhibitor (SP-2509 and clinical successor SP-2577, also known as seclidemstat), we observed significant synergy with JAK*i* (Ruxolitinib, 0–10 uM) (Fig. 6B) and with the BCL2 inhibitor (ABT-199, 0–600 nM) (Fig. S8C) in selectively reducing the growth of LOUCY ETP-ALL cells but not the mature T-ALL JURKAT cells. The scaffolding LSD1 inhibitor SP-2509 not only affected the H3K4 demethylase activity, as did GSK-LSD1*i* and ORY-1001 catalytic LSD1 inhibitors, but it also interferes with LSD1 in complex with other epigenetic regulators [28]. This scaffolding activity of LSD1 plays an important role in diverse cancer types [29, 30]. Indeed, previous reports have demonstrated that treatment with scaffolding LSD1 inhibitors can disrupt the LSD1 interaction with CoREST and GFI1/1B, thereafter inducing expression of myelo-monocytic differentiation-associated markers (CD86 and CD11b) and acquisition of morphologic differentiation features in AML cells [28, 31–33]. Interestingly, in our context, the scaffolding SP2509 inhibitor treatment led immature LOUCY ETP-ALL cells to increase expression of CD3ε a mature T cell marker (Fig. 6C). These increases in CD3ε expression in ETP-ALL cells may reflect enhanced differentiation and was only observed with SP2509 and not with GSK-LSD1*i* and was not modified in the presence of Ruxolitinib/JAK*i* within a 72-hour timeframe (Fig. 6D). This result highlights for the first time the potential importance of the scaffolding activity of LSD1 in human ETP-ALL leukemic cells.

### Exploring the therapeutic potential of targeting JAK/STAT pathway to sensitize human ETP-ALL to LSD1*i* (SP2509)

**Fig. 7:**
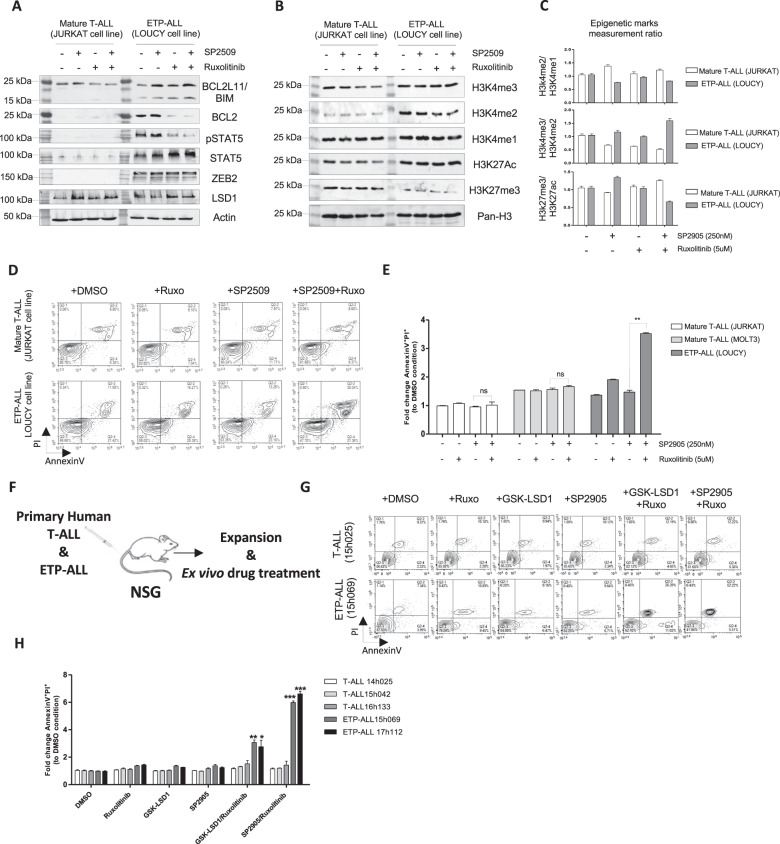
Exploring the therapeutic potential of LSD1 steric inhibitor SP2509 and JAKi Ruxolitinib drug combinations in human mature T-ALL and ETP-ALL in vitro. **A** Western blot assessing the protein expression of anti-apoptotic BCL2, pro-apoptotic BIM (*Bcl2l11*), phosphoSTAT5(nuclear), total STAT5, ZEB2 and LSD1 and (**B**) Epigenetic marks in mature JURKAT T-ALL cell line and LOUCY ETP-ALL cell line cultured with Ruxolitinib (5uM) with/without SP2509 (250 nM). **C** The measurement of the epigenetic marks illustrated in Sup Fig. 4C, and the panel present the ratios H3K4me2:H3K4me1, H3K4me3:H3K4me2 and H3K27me3:H3K27ac to assess the dynamics of methylation and acetylation of histones in human mature JURKAT T-ALL and LOUCY ETP-ALL cell lines. **D** AnnexinV/PI analysis of apoptosis in human mature JURKAT and MOLT3 T-ALL cell lines and human LOUCY ETP-ALL cell line, and (**E**) Normalized AnnexinV/PI^+^ measurement for each cell line treated with Ruxolitinib (5uM) with/without SP2509 (250 nM). **F** Schematic of expansion of primary human T-ALL and ETP-ALL in NSG mice following by ex vivo drug treatment in vitro. **G** Representative AnnexinV/PI flow cytometry results of primary T-ALL (15h025) and ETP-ALL (15h069) for control (DMSO), single drug (Ruxo, GSK-LSD1, SP2509) and combination drug exposure (GSK-LSD1/Ruxo, SP2509/Ruxo). **H** Normalized AnnexinV/PI^+^ measurement for each primary T-ALL (*N* = 3) and ETP-ALL (*N* = 2) patient sample treated with Ruxolitinib (5uM) with/without GSK-LSD1 (100 nM) and SP2509 (250 nM). Experiments were performed in triplicate. *p < 0.05, **p < 0.01, ***p < 0.001.

To further investigate the synergy of the scaffolding LSD1 inhibitor SP2509 with JAK*i* (Ruxolitinib) regarding the regulation of the intrinsic apoptosis pathway in human ETP-ALL, we performed western blot of pro- and anti-apoptotic proteins. As shown in Fig. 7A, the treatment with this drug combination efficiently reversed the expression of anti-apoptotic BCL2 protein and pro-apoptotic BIM protein in LOUCY ETP-ALL cell line expressing ZEB2. In contrast no changes were noticed in mature JURKAT T-ALL cell line.

Most interestingly, the global epigenetic marks showed an aberrant increase of H3K4me2 in LOUCY ETP-ALL compared to mature JURKAT T-ALL (Fig. S8D) similar to our previous observations in mouse ZEB2^Tg^ ETP-ALL. In addition, the treatment with the combination of SP2509 with Ruxolitinib significantly reduced the H3K4me2/1 marks without affecting the accumulation of active H3K4me3 or H3K27ac marks, that is opposite to what was observed in mature JURKAT T-ALL cells (Fig. 7B, C). Of note, the reduced methylation of H3K27 (H3K27me3-Fig. 7B) observed in LOUCY ETP-ALL is the consequence of the inactivating mutation in EZH2, which is the enzymatic subunit of the PRC2 complex that methylates the H3K27 [34]. In fact, mutations affecting the activity of PRC2 complex are present in 40% of ETP-ALL patients and highly associated with activating mutations of the IL7R/JAK/STAT pathway [35] which gives relevance to using human LOUCY ETP-ALL cell line to model to study the biology of ETP-ALL leukemia [4, 36].

### Pre-clinical validation of drug combination to target human ETP-ALL in vitro and in vivo

We next investigated the therapeutic potential of this combinatorial drug by carrying out Annexin V/PI analysis to assess their effects on apoptosis on human LOUCY ETP-ALL in vitro. Targeting JAK/STAT pathway with Ruxolitinib increased the sensitivity of human LOUCY ETP-ALL to SP2509 which resulted in increased apoptosis of human LOUCY ETP-ALL. In contrast, no synergistic effect was observed when JURKAT and MOLT3 mature T-ALL cell lines were treated in the same conditions (Fig. 7D, E). Moreover, the treatment with BCL2*i* (ABT-199) in combination with SP2509 also enhanced efficiently and specifically the apoptosis response of human LOUCY ETP-ALL (Fig. S8E, F).

To determine the relevance of these in vitro experiments on primary acute leukemia, we obtained primary human patient T-ALL (*N* = 3) and ETP-ALL (*N* = 3) bone marrow biopsy samples and expanded these cells in vivo in NOD.Cg-*Prkdc*^*scid*^ *Il2rg*^*tm1Wjl*^/SzJ (NSG) immunocompromised mice for up to 6 months (Fig. 7F). Expanded human T-ALL (*N* = 3) and ETP-ALL (*N* = 2) samples were then exposed to single inhibitors against LSD1 (GSK-LSD1, SP2509), JAK1/2 (Ruxo) or BCL2 (ABT-199) or combinations of GSK-LSD1/Ruxo, GSK-LSD1/ABT-199, SP2509/Ruxo or SP2509/ABT-199. Like mature mouse T-ALL and human JURKAT cell lines, no effects on apoptosis of single agents or combination therapy were observed in any of the three primary patient derived T-ALL cells in vitro (Fig. 7G, H, Fig. S8 G, H). Unlike the human LOUCY cell line, both primary patient ETP-ALL samples were sensitive to GSK-LSD1/Ruxo and GSK-LSD1/ABT-199 combination therapy (Fig. 7G, H, Fig. S8G, H) similar to primary mouse Zeb2^Tg^ ETP-ALL and cell lines, but here the highest levels of apoptosis were observed in the primary ETP-ALL samples that were treated with SP2509/Ruxo (Fig. 7G, H) or SP2509/ABT-199 combinations (Fig. S8G, H).

**Fig. 8:**
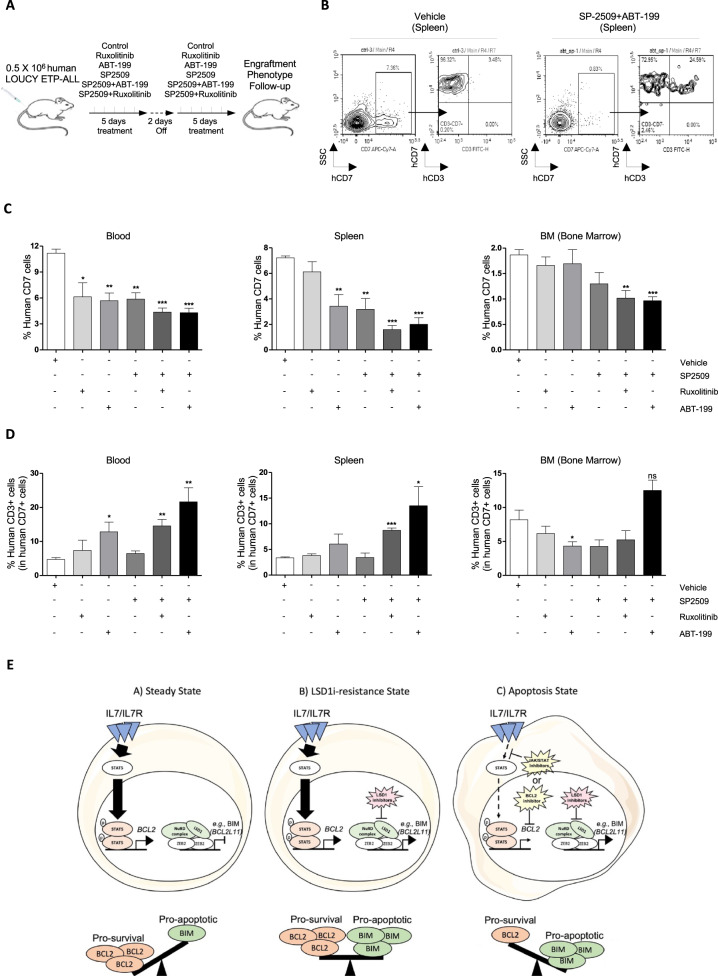
Exploring the therapeutic potential of LSD1 steric inhibitor SP2509 and JAKi Ruxolitinib drug combinations in human ETP-ALL cells in vivo. **A** Experimental strategy. NSG mice were engrafted with 0.5 × 10^6^ of human ETP-ALL LOUCY cell line and treated by gavage or intraperitoneal (for SP2509) with vehicle control, SP2509 (25 mg/kg/every two days) and/or Ruxolitinib (30 mg/kg/once a day), ABT-199 (25 mg/kg/ once a day) for 2 consecutive weeks. **B** Engraftment of human ETP-ALL LOUCY cells was assessed in blood, spleen and bone marrow (BM) of treated recipient mice by using the human CD7 T cell marker, **C** Confirmation of the high synergy of the drug combination LSD1i (SP2509) with JAK*i* (Ruxolitinib) or BCL2*i* (ABT-199) in efficiently reducing the growth of human ETP-ALL LOUCY cells in vivo, (**D**) Enhancement of LOUCY cell differentiation in vivo*. n* = 4 mice/group. *p < 0.05, **p < 0.01. ***p < 0.001. (E) Model. Left panel- In steady state ETP-ALL, the high-activity of IL7/IL7R increases the expression of the pro-survival proteins e.g., BCL2. Middle panel- Treatment with LSD1 inhibitors (LSD1i) increases the expression of pro-apoptotic proteins e.g., BIM (BCL2L11) but is not sufficient to reverse the activity of pro-survival proteins e.g., BCL2. Right panel- Simultaneous inhibition of JAK-STAT pathway or BCL2 protein in combination with LSD1 inhibitors induces an efficient therapy against malignant ETP-ALL leukemia cells.

We next investigated the effect of drug combination in human ETP-ALL in vivo. NSG mice engrafted with human ETP-ALL LOUCY cells were treated with SP2509 (25 mg/kg/every two days) [28] combined or not with Ruxolitinib (30 mg/kg/ once a day) [27] or ABT-199 (25 mg/kg/ once a day) [37] for 2 consecutive weeks (Fig. 8A). Levels of human CD7^+^ leukemic blasts in mouse blood, spleen and bone marrow confirmed the efficiency of LSD1*i* (SP2509) combined with JAK*i* (Ruxolitinib) or BCL2*i* (ABT-199) to reduce the leukemic cell growth in vivo compared to either drug alone (Fig. 8B, C). Interestingly, the CD3ε mature T cell marker expression was enhanced in vivo in immature human ETP-ALL LOUCY cells from blood and spleen, but not from the bone marrow confirming our previous observations with mouse ETP-ALL (Fig. 8B, D).

## Discussion

Early T-cell precursor acute lymphoblastic leukemia (ETP-ALL) is a relatively recently identified subtype of T-ALL with distinctive gene expression and cell marker profiles. ETP-ALL is derived from thymic cells at an early T-cell precursor (ETP) [4] stage and share some gene expression features with hematopoietic stem cell and myeloid progenitor cells [2]. From a clinical perspective, ETP-ALL patients show poor responses to chemotherapy and exhibit a very high risk of relapse. Despite an overall complete response (CR) rate of 73% after treatment with combination chemotherapy, the median overall survival for ETP-ALL patients is approximately 20 months [10]. Based on these clinical outcomes, the actual major challenge in the treatment of ETP-ALL patients is to overcome the drug-resistance process and to understand the molecular mechanisms by which malignant cells escape chemotherapeutic treatment or monotherapies [38].

ZEB2 is a transcriptional regulator of the epithelial to mesenchymal transition (EMT) process and previous work has demonstrated its involvement in the leukemogenesis of ETP-ALL [8, 11]. In addition, its expression is usually associated with enhanced cancer stemness programs and chemo/radiation resistance of solid tumours [39]. By using a proteomics approach, we unveiled the interaction of ZEB2 with epigenetic NuRD complex including LSD1 in human and mouse ETP-ALL [11]. Through this present study, we have demonstrated the specific role played by ZEB2 and NuRD complex (through its enzymatic demethylase subunit LSD1/KDM1A) in the regulation of the intrinsic apoptosis pathway, which reiterates the therapeutic potential of targeting epigenetic co-factor LSD1 to cure ETP-ALL.

Furthermore, selective analysis of epigenetic marks associated with LSD1 in ETP-ALL and mature T-ALL show a divergence of their epigenetic landscapes. Indeed, epigenetic abnormalities are common and critical for human cancers, especially hematopoietic malignancies [40]. Many cancer-associated epigenetic alterations promote tumorigenesis by impairing the differentiation and survival mechanisms of normal cells. These epigenetic alterations observed in cancer cells are a result of a competition between several epigenetic complexes. For instance, in myeloid leukemia, LSD1 (H3K4 demethylase subunit of NuRD complex) plays an important oncogenic role through its repressive activity. Interestingly, its inhibition using the LSD1 scaffolding inhibitor (OG86) increased the binding of H3K4 methyltransferase MLL4 (an enzymatic subunit of COMPASS complex) to the promoter of BAX and activated its expression in leukemic cells, suggesting that LSD1/NuRD complex and COMPASS complex might directly compete for binding to the promoters of differentiation-related genes and tumor suppressors [31]. Thus, the partial inactivation of H3K4 methyltransferase COMPASS complex (through the knockout of UTX [41] subunit, named herein COMPASS-like complex) in hematopoietic progenitor cells leads to the global accumulation of H3K4me2, and targeting LSD1 (demethylase subunit of NuRD complex) with the scaffolding inhibitor (SP2509) restored the balance in H3K4 methylation and led to passive increases of the active mark H3K4me3 by the COMPASS-like complex that correlates with differentiation and the active expression of specific differentiation-related or tumor suppressor genes (*see* Fig. 5a, b in [15]). Indeed, this global reduction in active H3K4 methylation is due to the increased turn-over of H3K4me2/1 that is elicited by the LSD1/NuRD complex, and also through its scaffolding (-demethylase independent) activity that prevents the binding of COMPASS complex which consequently reduces the accumulation of the active marks H3K4me3 in the gene promoters [15]. Regarding the role of the epigenetic mark H3K4me2 in regulation of gene expression, it has been demonstrated in multipotential hematopoietic cell lines the existence of a population of genes in a unique epigenetic state defined by the presence of di-methylation, but not tri-methylation, of H3K4 (H3K4me2^+^/H3K4me3^-^). These genes are transcriptionally poised and are specifically activated in a lineage-specific manner such as upon erythroid differentiation [13]. Functionally, the enrichment of epigenetic mark H3K4me2 in gene promoters is associated with recruitment of HDACs (subunit of NuRD complex) to suppress cryptic internal transcriptional initiation [42, 43].

In the cancer context, increased activity of the LSD1/NuRD complex through oncogenes such us ZEB2 could lead to the same process of gene repression by poising gene expression and blocking differentiation at an immature and cancer stem cell-like state. Indeed, the accumulation of H3K4me2 observed in human and mouse ETP-ALL was decreased by targeting LSD1 (with GSK-LSD1 or SP2509) in combination with JAK*i*, which led to a global reverse of the H3K4me3:H3K4me2 and H3K4me2:H3K4me1 ratios (Fig. 3C, Fig. 7C). In addition, combination therapy induced the differentiation and the reactivation of certain therapeutic relevant genes such as *Bcl2l11* (BIM). Moreover, this accumulation of H3K4me2 at binding site of ZEB2/LSD1 in *Bcl2l11* promoter, associated with its repression, was already documented previously in other cell contexts (e.g. muscle stem cells), whereby binding of the MyoD transcription factor to the promoter of *Patz1*, a stem cell expressed-gene, led to the accumulation of H3K4me2 and consequently reversed the ratio H3K4me3:H3K4me2 (active vs repressed) that correlated with a decrease of *Patz1* expression in differentiated muscle cells [12].

The therapeutic strategy of reactivating the expression of *BCL2L11* (BIM) in cancer cells, in order to induce their apoptosis, has been explored for decades in previous studies [44]. In the acute lymphoblastic leukemia context, different mechanisms of *BCL2L11* (BIM) regulation have been identified [45]. For instance, in mature T-ALL, the repression of *BCL2L11* (BIM) expression is ensured by the EZH2/PRC2 complex through the transcription factor HES1 that is known to be regulated by NOTCH1 singling pathway in mature T-ALL. Targeting this pathway with γ-secretase inhibitor (NOTCH1 pathway inhibitor) releases the repression of *BCL2L11* expression and consequently reverses the resistance of mature T-ALL to chemotherapy with Dexamethasone [46]. The mutation of PCR2 complex in more than 40% of ETP-ALL patients [36] could invalidate this therapeutic strategy, which may also argue for the importance of our study regarding this new repression mechanism of *BCL2L11* (BIM) by LSD1/NuRD complex and ZEB2 in the ETP-ALL context, and that could confer them a selective sensitivity to LSD1 inhibitors.

More globally, the application of epigenetic drugs in the clinic is gaining more interest, and this is due to their increasing efficacy and specificity [47]. Targeting LSD1/NuRD complex with available epigenetic drugs (LSD1*i*) to reactivate the expression of pro-apoptotic BIM protein could offer better clinic outcomes for ETP-ALL patients. Mechanistically, the increased expression of BIM protein antagonizes the interaction of anti-apoptotic members such as BCL2 with multidomain proteins BAX, BAK and BOK, which promotes mitochondrial outer membrane permeabilization (MOMP) and the release of cytochrome *c* that reactivates the apoptosis process^47^ (Fig. 2C). This illustrates the importance of BCL2/BIM ratio for any efficient therapy targeting the viability of ETP-ALL [48].

Intriguingly, in mouse and human ETP-ALL, the treatment with LSD1*i* led not only to increase expression of BIM, but also to slight increases the BCL2 expression that is already highly expressed, which keeps the ratio BCL2/BIM (anti- /pro-apoptotic) similar in untreated cells (Fig. 2E, F and Fig. 3A). Indeed, this increased expression of BCL2 under treatment with LSD1*i* could be associated to the low presence of NuRD complex in active genes such as BCL2 to “fine tune” their expression (Fig. 3A) [49, 50]. Moreover, our results shed light on one facet of the resistance process of ETP-ALL to LSD1*i* that could explain the inefficiency of LSD1*i* as monotherapy [11], and highlights the need for targeted therapy against BCL2 expression in ETP-ALL context to potentialize the therapeutic activity of LSD1*i* in ETP-ALL.

Through our work, we confirmed that STAT5b that is downstream of IL7/IL7R signaling increases the expression of BCL2 in mouse and human ETP-ALL. Indeed, previous work has demonstrated the role played by the hyper-activation of IL7/IL7R signaling in the leukemogenesis of ETP-ALL [6, 51]. Our results confirmed that STAT5b bound the enhancers associated to *Bcl2* locus and the measurement of epigenetic marks in these regions by ChIP qPCR demonstrated an increase in active mark H2K27ac that occurs by the recruitment of its coactivators like the acetyltransferase p300 [52]. Indeed, the inhibition of JAK/STAT pathway with small drugs (e.g. Ruxolitinib) decreased the phosphorylation and consequently the translocation of STAT5b to the nucleus which reduced its transcriptional activity at *Bcl2* gene.

The combination of LSD1*i* with JAK/STAT pathway inhibitor (JAK*i*, Ruxolitinib) reversed efficiently the expression ratio of BCL2/BIM (anti-/pro-apoptotic) in human and mouse ETP-ALL. The synergy between LSD1*i* and JAK*i* was highly active to specifically compromise the growth and the viability of ETP-ALL in vitro and in vivo. Indeed, targeting directly BCL2 protein with ABT-199 in combination with LSD1*i* phenocopied the effects obtained with JAK*i* in ETP-ALL, which confirmed the central role of JAK/STAT/BCL2 molecular axis in the resistance process of human and mouse ETP-ALL to LSD1*i*.

Finally, by testing the available LSD1 inhibitors in human ETP-ALL, we noticed that scaffolding LSD1 inhibitor (SP-2509 and clinical successor SP-2577, also known as seclidemstat) has greater activity on its own as well as more synergy with JAK/STAT pathway inhibitors than the catalytic LSD1 inhibitors (e.g. GSK-LSD1, ORY-1001), which highlights in addition to the demethylase activity, the scaffolding role of LSD1 in human ETP-ALL to maintain the repression of ZEB2 target genes such as pro-apoptotic genes (e.g. *BCL2L11*(BIM). Previous studies have highlighted the importance of scaffolding activity of LSD1 in diverse cancer types [29, 30, 53, 54] and confirmed our finding that the catalytic inhibition of LSD1 is insufficient as a therapeutic strategy in human ETP-ALL [29, 30, 55], which needs further study. Recently, the SP2509 LSD1 inhibitor has been demonstrated to impact on JAK/STAT3 signaling [56] as well as pro-survival protein expression/stability of BCL2 and MCL1 respectively [57] and may explain why the SP2509 inhibitor is more active in ETP-ALL than the GSK-LSD1 compound.

Overall, our results highlight the resistance mechanism of ETP-ALL to LSD1*i* monotherapy and provides a new anti-leukemia combination therapy approach for ETP-ALL that could be readily assessed in clinical trials (Fig. 8E), especially given the fact that we tested only targeted drugs that are FDA approved and already used in the clinic as monotherapies [14, 58–60].

## Methods and materials

### Cell lines and cell culture

Primary mouse thymic lymphoma cell lines from Lck-Cre, R26-Zeb2^Tg/Tg^, p53^Fl/Fl^ background were generated according to the previously published protocols [11]. All mouse derived cell lines were cultured in RPMI medium supplemented with 15% heat-inactivated fetal bovine serum (Sigma), penicillin (100 U ml^−1^)-streptomycin (100 μg ml^−1^), 2 mM L-glutamine (Gibco), 0.05 mM 2-mercaptophenol, 15 ng ml^−1^ recombinant IL-7 (Peprotech) and incubated at 37 °C with 5% CO_2_ and 95% humidity at the concentration of 0.3 × 10^6^ cell per ml. For cell survival and proliferation assays, the cells were seeded at a concentration of 0.5 × 10^6^ cell per ml in medium with or without 15 ng ml^−1^ IL-7 and incubated at 37 °C, 5% CO_2_ and 95% humidity and treated with increased concentration of LSD1i and/or Ruxolitinib, ABT-199. the cells were counted after 24 h, 48 and 72 h post-treatment with TC20 Automated Cell Counter (BIO-RAD). Lentiviruses expressing an anti-ZEB2 shRNA or a control shRNA (Anti-luciferase) (LT3GECIR; Addgene#111178) [61] (see the Primer list table for sequence) were prepared as previously described [62] and used to infect cell lines. the cell lines were infected by spinoculation (800 g for 30 min) at a multiplicity of infection (MOI) of 50 in the presence of 8 μg/mL hexadimethrine bromide (Polybrene, Sigma). The infection was performed twice at 24-h intervals, and the cells were harvested 72 h after the first infection. Flow cytometry was used to assess the GFP expression by transduced cells at day 1 (post-induction with DOX, 500 ng/ml) and day 10 of culture.

The human cell lines LOUCY, JURKAT and MOLT3 were obtained from the Leibniz-Institute DSMZ-German Collection of Microorganisms and Cell Cultures (Braunschweig, Germany). All human cell lines were cultured in RPMI 1640 (ThermoFisher Scientific) supplemented with 10%, 1% Pen/Strep, and 1% l-glutamine at 37 °C in 5% CO_2_. All cell lines were routinely analysed for the presence of mycoplasma. For cell survival and proliferation assays, the cells were seeded at a concentration of 0.3 × 10^6^ cell per ml at 37 °C, 5% CO_2_ and 95% humidity and treated with increased concentration of LSD1i and/or Ruxolitinib, ABT-199. the cells were counted after 24 h, 48 and 72 h post-treatment with TC20 Automated Cell Counter (BIO-RAD).

### RT‐qPCR and ChIP-qPCR

For real-time quantitative PCR (RT-qPCR), RNA was extracted using the illustra RNAspin Mini Kit (GE Healthcare) and reverse transcribed with the perfectStart Grenn qPCR super Mix kit (Transgenbiotech). The cDNA was PCR amplified in triplicate using the Fast SYBR green dye (ThermoFisher Scientific) on the Applied Biosystems QuantStudio 7 Flex Real-Time PCR System. Relative expression was determined using the ACTB and TUBB5 as internal control. For the sequences of primers check the Primer list table. For the qPCR heatmap, we mapped the log2 gene expression using the QCanvas software (version 1.21).

For ZEB2, LSD1, HDAC1, STAT5, H3K4me1, H3K4me2, H3K4m3, H3k27Ac and H3K27me3 ChIP were performed from T-ALL (WT-1) and ETP-ALL (TG-50) cells as previously described [63] using the antibodies listed in Abs table. For quantitative PCR (qPCR), chromatin-immunoprecipitated DNA was quantified by real-time qPCR with SYBR Green using a standard curve generated with genomic DNA and was normalized by dividing the amount of the corresponding target in the input fraction.

### Flow cytometry

The phenotype, enframement of mouse and human cells were performed on a Novocyte flow cytometer using human specific mouse monoclonal antibodies provided in Abs table. Analyses of raw data were performed using NovoExpress software (Agilent).

### Flow cytometric analysis of viability and apoptosis

Following 2- or 3-days’ incubation with the indicated inhibitors, cell viability was assessed by flow cytometry. The cells were washed twice with cold PBS and incubated at room temperature in 1× binding buffer (10 mM HEPES, 140 mM NaCl, 2.5 mM CaCl_2_) containing AnnexinV-APC (BD) and Propidium Iodide (PI) using FITC Annexin V Apoptosis Detection Kit II (BD bioscience). Apoptotic cells were determined by flow cytometry within 1 h of staining.

### Measurement of mitochondrial membrane potential (ΔΨm)

Mitochondrial membrane potential (ΔΨm) was determined by the JC-1 probe method according to the manufacturer’s protocol (JC-1 Mitochondrial Membrane potential Assay Kit, Cayman Chemical). In brief, after the treatment with indicated conditions, the cells were harvested, washed and stained with 10 μg/mL JC-1 at 37 °C for 30 min in the dark. Subsequently, stained cells were washed, resuspended, and subjected to flow cytometry analysis.

### Graphs and data analysis

All the graphs and statistical analyses were performed with PRISM (version 6.0.1). For the illustrative schema we used Servier Medical Art images. ImageJ software for Western blot quantification bands.

## Supplementary information

Supplementary Methods and Materials

Supplementary Table S1

Supplemenetary Table S2

## Data Availability

All data and reagents used in this publication are available upon request.
