# Supplementary material for: JAK/BCL2 inhibition acts synergistically with LSD1 inhibitors to selectively target ETP-ALL
Source: Leukemia. 2022 Oct 13;36(12):2802–16. doi: 10.1038/s41375-022-01716-9 (PMC9712096; doi:10.1038/s41375-022-01716-9)
Supplement: Supplementary file 1 — Supplementary Methods and Materials [file 41375_2022_1716_MOESM1_ESM.pdf]

Supplementary Materials for

JAK/BCL2 inhibition acts synergistically with LSD1 inhibitors to selectively target ETP-ALL

Aissa Benyoucef, Katharina Haigh, Andrew Cuddihy, Jody J. Haigh

Correspondence to: aissabenyoucef@gmail.com & jody.haigh@umanitoba.ca

This PDF file includes:

Materials

Methods

Figures. S1 to S6

Tables S1 to S2

Materials

1- Western blot Antibodies table

HDAC1 antibody (mAb)	https://www.activemotif.com	39531	1/500
Zeb2 antibody	https://www.novusbio.com	NBP1-82991	1/500
LSD1 Antibody (1B2E5)	https://www.novusbio.com	NB100-1762	1/1000
beta-Actin Antibody (AC-15)	https://www.novusbio.com	NB600-501	1/2000
STAT5	https://www.cellsignal.com	94205	1/1000
P-STAT5(Tyr694)	https://www.cellsignal.com	9351	1/1000
H3K27me3	https://www.emdmillipore.com	07-449	1/5000
anti-H3K4me3	https://www.abcam.com	ab8580	1/5000
anti-pan histone H3	https://www.emdmillipore.com	07-690	1/5000
anti-H3K27Ac	https://www.abcam.com	ab4729	1/5000
anti-H3K4m2	https://www.activemotif.com	39079	1/5000
anti-H3K4me1	https://www.abcam.com	ab8895	1/5000
In Vivo MAb anti-mouse IL-7Rα (CD127)	https://bxcell.com/	A7R34	100ug/ml

2- Flow cytometry Antibodies table

CD45	557748	https://www.bdbiosciences.com
CD7	564020	https://www.bdbiosciences.com
CD3	555340	https://www.bdbiosciences.com
PerCP anti-mouse/human CD11b Antibody	101229	https://www.biolegend.com
APC/Cyanine7 anti-mouse Ly-6G Antibody	127623	https://www.biolegend.com
PE anti-mouse CD3 Antibody	100205	https://www.biolegend.com
APC Rat Anti-Mouse CD4	561091	https://www.bdbiosciences.com
PerCP anti-mouse CD8a Antibody	100731	https://www.biolegend.com
APC/Cyanine7 anti-mouse/human CD44 Antibody	103027	https://www.biolegend.com
PE-Cy™7 Rat Anti-Mouse CD25	561780	https://www.bdbiosciences.com

3- Primer list table (please check the Excel file: Table S2)

Methods

Western blots:

Protein extraction and immunoprecipitation were performed essentially as described (Benyoucef et al 2016). For the Immunoblotting, protein extraction was prepared from 100,000 to 200,000 cells for SDS-PAGE (12%) and proteins were transferred to nitrocellulose membranes and probed with different antibodies, see Abs table. For the immunoprecipitation, briefly, the beads were cross-linked with Abs washed twice with 1M immunoprecipitation buffer (25 mM Tris at pH 7.9, 5 mM $MgCl_2$, 10% [v/v] glycerol, 0.1 % [v/v] NP40, 1 M KCl, 0.3 mM DTT, protease inhibitors), two washes with 150 mM immunoprecipitation buffer (25 mM Tris at pH 7.9, 5 mM MgCl2, 10% [v/v] glycerol, 0.1 % [v/v] NP40, 150 mM KCl, 0.3 mM DTT, protease inhibitors) and incubated with Nuclear extraction for ON. Bound proteins were eluted with 5% acetic acid, dried and resolved by SDS-PAGE. Proteins were then identified by Western blot and quantified with ImageJ software.

Bioinformatic analysis of ENCODE ChIP-seq data:

The high-throughput sequencing data were obtained from https://www.encodeproject.org STAT5b (ENCFF248XJW) and its control (ENCSR766WPH). KDM1A/LSD1 (ENCFF087TDC) and its control (ENCSR000AKY). ZEB2 (ENCSR004GKA) and its control (ENCSR173USI, ENCSR554FDZ). The Raw data were trimmed to remove low-quality reads and mapped to the NCBI build 37.1 (hg19) human reference genome using BWA (version 0.7.17.4). To detect regions of enrichment, we used model-based analysis of ChIP combined with massively parallel sequencing (MACS2 callpeak) version 2.1.1.20 using default values with a ctrl ChIP as a background and a Minimum FDR (0.05 q-value) cutoff for peak detection. The common and

unique peaks were defined by bedtools Intersect intervals (version 2.29.0). We used the plotHeatmap (Version 3.3.2.0.1) to create a heatmap for score distributions across LSD1, ZEB2 and STAT5 peak genomic regions. Association of common peaks (ZEB2, LSD1 and STAT5) with genes was performed with GREAT (http://great.stanford.edu/public/html/index.php) using the Basal plus extension setting. Check the GO biological process obtained with GREAT. The list of genes related to regulation of intrinsic apoptotic signaling pathway was illustrated by STRING (https://string-db.org).

Animal experimentation and handling.

All experiments were performed according to the regulations and guidelines of the Ethics Committee for care and use of laboratory animals at the University of Manitoba. The mouse (Mus musculus) cohorts used in these experiments were sibling littermates and maintained. Both male and female mice were included in the various analyses and ranged in age from 3 to 60 weeks.

For the transplantation experiments, primary thymic tumours (Lck-Cre, R26-Zeb2$^{Tg/Tg}$, p53$^{Fl/Fl}$) were dissected under aseptic conditions. Cell was prepared in sterile PBS and the indicated cell numbers were intravenously injected in 6- to 10-week-old total body irradiated C57BL/6J wild-type recipient mice (Charles River). A similar approach was used for the transplantation of LOUCY cells into NSG mice (Charles River). For drug treatments, after 3-7 days post-injection, the engraftment was measured routinely (5-10%) in peripheral blood, and randomly the mice were treated with SP2509 (Cedarlane lab, CA) prepared in solvent buffer (20% PEG-40, 20% dimethyl sulfoxide, 60% sterile water) by intra-peritoneal injection. ABT-199 (Cedarlane lab, CA) prepared in 2.5%dimethyl sulfoxide, 50% polyethylene glycol 300, 5% Tween 80, and 42.5% of 0.5% methyl cellulose in water, administrated in mice by oral gavage. For Ruxolitinib phosphate (Chemscene. USA) prepared in vehicle (20% Captisol in 58 mM citrate buffer) twice daily by oral gavage. For GSK-LSD1(Chemscene. USA) prepared in vehicle (sterile NaCl 0.9%) administrated in mice by oral gavage.

Patient-derived xenograft models in NSG mice.

NSG (NOD.Cg-Prkdc<scid>Il2rg<tm1Wjl>SzJ) male/female mice (8–10 wk of age) were purchased from The Jackson Laboratory and maintained under sterile conditions at the University of Manitoba Animal Care Facility. Leukemic blasts from patients (characterized and provided by The Quebec Leukemia Cell Bank) were expanded in NSG mice by transplanting 0.5×10^6 to 2×10^6 cells via intravenous injection. Engraftment of human leukemic blasts in the peripheral blood was assessed by FACS staining for human CD45 and human CD7 during a period of 1-6 months. Mice were euthanized when the engraftment of leukemic blasts was 5-30%, and BM and spleen cells were harvested. All the ETP-ALL and mature T-ALL cell patients are characterized and provided by The Quebec Leukemia Cell Bank (BCLQ) with the consent of all subjects involved.

Drug treatment of xenograft Patient-derived cells.

To ensure the high purity of blasts harvested from the BM of engrafted NSG mice, we used the EasySep™ FITC Positive Selection Kit II (StemCell Technologies, 17682) to sort the stained blasts with the anti-human CD7 FITC. For the drug treatment in vitro, from each condition, the sorted blasts were cultured on OP9 stromal cells expressing the NOTCH ligand Delta-like-1 (DL1) as previously described (Benyoucef et al. 2015). Briefly, $1x\ 10^5$ cells from each sample were plated in α minimum essential medium with 10% fetal calf serum (StemCell Technologies, 06450) and 10% serum (Sigma-Aldrich, H4522) supplemented with 50 ng/mL hSCF cytokine, 20 ng/mL Flt3-L cytokine, 20 nM insulin, 10ng/mL IL-7, and 100 U/mL penicillin and 100 mg/mL streptomycin. All human cytokines are provided by peprotech Canada. The cells are treated for 5 days with DMSO (as control condition), Ruxolitinib alone (5uM), GSK-LSD1 alone (100nM), SP-

2905 alone (250nM), ABT-199 alone (25nM), or with drug combination indicted in the Figure 7 and S8. The apoptosis of blasts after treatment was assessed as mentioned previously.

Human mRNA expression in primary human samples.

We used the mRNA expression data in human patient ETP-ALL (n=12) vs mature T-ALL (n=40) samples previously published (Zhang J et al, 2012, Gutierrez A et al. 2011), and publicly available at Gene Expression Omnibus-NCBI, GSE28703.

Figure S1.

(**A**) Western blot (WB) analysis of uncrosslinked immunoprecipitation of 3XFLAG tagged ZEB2 performed in T-ALL ($P53^{-/-}$) and ETP-ALL ($ZEB2^{tg/tg}$ $P53^{-/-}$) cell line nuclear extracts (NEs) with Abs against endogenous ZEB2, LSD1 and STAT5. (**B**) ZEB2 knockdown (KD) with 3 independent ShRNAs measured by Western blot. (**C**) Cell growth measured by the fold change number cells (D10/D1) after the ZEB2 knockdown in T-ALL ($P53^{-/-}$ -WT-1) and ETP-ALL ($ZEB2^{tg/tg}$ $P53^{-/-}$-TG-50) from 3 independent experiments (**D**) Representative cytometry plots of the experiments used to measure the cell growth of T-ALL ($P53^{-/-}$-WT-1) and ETP-ALL ($ZEB2^{tg/tg}$ $P53^{-/-}$-TG-50) cell line (illustrated in (**C**)) after the efficient KD of ZEB2 with ShZeb2#2 & ShZeb2#3. Representative flow cytometry blots of ShZeb2#2 & ShZeb2#3 that were used to assess the growth of transduced GFP^{+} T-ALL ($P53^{-/-}$-WT-1) and ETP-ALL ($ZEB2^{tg/tg}$ $P53^{-/-}$-TG-50) cells at day 1 and day 10 of the culture (**E**) Viability assays in the T-ALL ($P53^{-/-}$) cell lines and ETP-ALL ($ZEB2^{tg/tg}$ $P53^{-/-}$) cell lines treated for 48h with (open circles) or without (black squares) IL7 cytokine (10ng/ml) plus increasing concentrations of ORY-1001 or (**F**) with SP2509. All experiments were performed in triplicate. *p< 0.05; **p< 0.01, ***p< 0.001.

Figure S2.

Exploring the transcriptional role of ZEB2, LSD1 and STAT5a in leukemic context by using publicly available high throughput sequencing data (ENCODE Project) (**A**) Average profile and heat map for LSD1, ZEB2 and STAT5b ChIP–seq in same leukemic context (K562) data obtained from ENCODE project 3/4. Metagene plot ± 5 kb is shown. $P < 0.05$ (**B**) Venn diagram of overlapping peaks bound by LSD1, ZEB2 and STAT5b protein using significance threshold at $P < 0.01$. (**C**) analysis the genome binding distribution of the common peaks (ZEB2-LSD1-STAT5b). (**D**) GO analysis of common-bound genes (LSD1/ZEB2/STAT5b) classified by the Binomial *p* value. (**E**) STRING interaction network of the genes involved in regulation of intrinsic apoptosis signaling pathway (highlighted by * in **D**) and potentially regulated by LSD1, ZEB2 and STAT5b (n=109 genes).

Figure S3.

A) RNA expression of the anti-apoptotic proteins (BCL2, BCL2L1, MCL1) and anti-apoptotic protein BIM (BCL2L11) in T-ALL (n=40) and ETP-ALL (n=12) patients. **B**) Time course of IL7 stimulation, after 6h without stimulation, the mouse T-ALL (WT-1) and ETP-ALL (TG-50) was stimulated with 10ng/ml of IL7 and the RNA expression of *Bcl2*, *Bcl2l1*, *Mcl1* and *Bcl2l11* (BIM) genes was measured at different time point (0, 30, 60 and 120 min). $n = 3$. (*) $P < 0.05$; (**) $P < 0.01$; (***) $P < 0.001$. **C**) Assessing the role of IL7R in the regulation of the anti-apoptotic proteins (BCL2, BCL2L1, MCL1) in T-ALL (WT-1) and ETP-ALL (TG-50) using the Ruxolitinib (50ng/ml) or the anti-IL-7Rα (100ug/ml).

Figure S4.

(**A**) Measuring the cell growth of mouse T-ALL (WT-1) and ETP-ALL (TG-50) after treatment with increasing concentration of specific inhibitor of MCL1 (AZD5991), inhibitor of BCL2L1 (WEHI-

539), or inhibitor of BCL2 (ABT-199) in presence or absence of GSK-LSD1 inhibitor (100nM) n = 3. (*) $P < 0.05$; (**) $P < 0.01$. **B&C**) apoptosis measurement after 48h of treatment with MCL1 inhibitor (AZD5991, 10nM), BCL2L1 inhibitor (WEHI-539, 10nM) or BCL2 inhibitor (ABT-199, 10nM) in combination or not of GSK-LSD1 inhibitor (100nM). n = 3. (*) $P < 0.05$; (***) $P < 0.001$.

Figure S5.

UCSC genome browser tracks showing ChIP–seq of LSD1, ZEB2 and STAT5 in representative Bcl2, Bcl2l11 and CD8a loci in leukemic context (K562) using the ENCODE project data (*See Sup Figure 2A*). The sonication optimization (~90 cycles our optimal condition) (**B**) and the validation by western blot of epigenetic marks antibodies used in ChIPqPCR experiments (**C**) in T-ALL ($P53^{-/-}$-WT-1) and ETP-ALL ($ZEB2^{tg/tg}$ $P53^{-/-}$-Tg-50) cell lines. (**D**) Quantification of western results for global methylation marks in T-ALL and ETP-ALL cells.

Figure S6.

Assessing the toxicity and the tolerability of combination therapy for GSK-LSD1 and Ruxolitinib or ABT-199 drug *in vivo.* (**A**) Experimental strategy. Healthy C57BL/6J mice were treated by gavage with vehicle control, GSK-LSD1 (1.5mg/kg/day) and/or Ruxolitinib (30mg/kg/ twice a day) for 2 consecutive weeks. (**B**) the impact of treatment with single or combined drugs on different hematopoietic cells using the cell surface markers(Ter119 (Erythroids), Ly6G (Neutrophils), Thy1.2 (T cells), CD11b (Mono/eosinophils), CD41 (Megakaryocytes) and B220 (B cells) (**C**) and assessed in different tissue by flow cytometry assays that revealed slight increases in mono/eosinophils in spleen tissue but overall, no sign of toxicity or intolerability was observed during and after the 2 weeks of treatment. n = 4 mice/group *$p < 0.05$.

Figure S7.

(**A**) RNA expression of the known oncogenes (LYL1, LMO2, HHEX) and ZEB2 in T-ALL (n=40) and ETP-ALL (n=12) patients. (B) ZEB2 protein measurement with western blot in human ETP-ALL cell line (LOUCY) transduced with shCtrl or 3 independent shRNA against ZEB2. (**C**) Flow cytometry analysis of GFP+ cells over time of transduction human ETP-ALL LOUCY cell line with ShCtrl or 3 independent shRNA_ZEB2 and (**D**) measuring the % of GFP^+ cells at Day 1 and Day 7 of the culture $n = 3$. (*) $P < 0.05$; (**) $P < 0.01$. (**E**) Assessing the growth of GFP^+ LOUCY cells using fluorescent microscopy at Day 1 and Day 12 (400× magnification [0.4 mm]).

Figure S8.

Drug synergy analyzed by using Combenefit software reveled the absence of synergy between the methylase LSD1 inhibitors (**A**) GSK-LSD1 (high-dose) or (**B**) ORY-1001) with Ruxolitinib. (**C**) the high synergy of scaffolding LSD1 inhibitor (SP2509) with BCL2*i* (ABT-199) to compromise cell growth of human ETP-ALL LOUCY cell line but not JURKAT cells. (**D**) Measurement of epigenetic marks from 3 independent experiments performed in human mature T-ALL JURKAT and ETP-ALL LOUCY cell lines cultured with Ruxolitinib (5uM) with/without SP2509 (250nM) assayed by western blot analysis and protein quantification compared to internal loading controls. $n = 3$ *p< 0.05; **p< 0.01. (**E**) Using the AnnexinV/PI assay to measure the apoptosis of human mature T-ALL JURKAT and MOLT3 cell lines and unique ETP-ALL LOUCY cell line, and (**F**) Normalized measurement of AnnexinV/PI^+ cells in each cell line treated with ABT-199 (10nM) with/without SP2509 (250nM). n =average of 3 experiments **$p < 0.01$. (**G**) Representative AnnexinV/PI flow cytometry results of primary T-ALL (15h025) and ETP-ALL (15h069) for control (DMSO), single drug (Ruxo, GSK-LSD1, SP2509) and combination drug exposure (GSK-LSD1/Ruxo, SP2509/Ruxo). (**H**) Normalized AnnexinV/PI^+ measurement for each primary T-ALL (N=3) and ETP-ALL (N=2) patient sample treated with ABT-199 (25nM)

with/without GSK-LSD1 (100nM) and SP2509 (250nM). Experiments were performed in triplicate. **p<0.01, ***p< 0.001.

Figure S9.

Uncropped western blot analysis of murine T-ALL (WT-1) and ETP-ALL (TG-50) cell lines for effects of GSK-LSD1, ruxolitinib and combination treatment on H3 epigenetic marks.

Figure S10.

Uncropped western blot analysis of human T-ALL (JURKAT) and ETP-ALL (LOUCY) cell lines for effects of SP2509, ruxolitinib and combination treatment on H3 epigenetic marks.

Table S1.

Supplementary table1 excel file. It contains the common ChIP peak locations. List of genes associated with common peaks. The results of the PANTHER, GREAT analysis, STRING intrinsic apoptosis genes of the common peaks.

Table S2.

The list of primers used in this study.

Figure S1

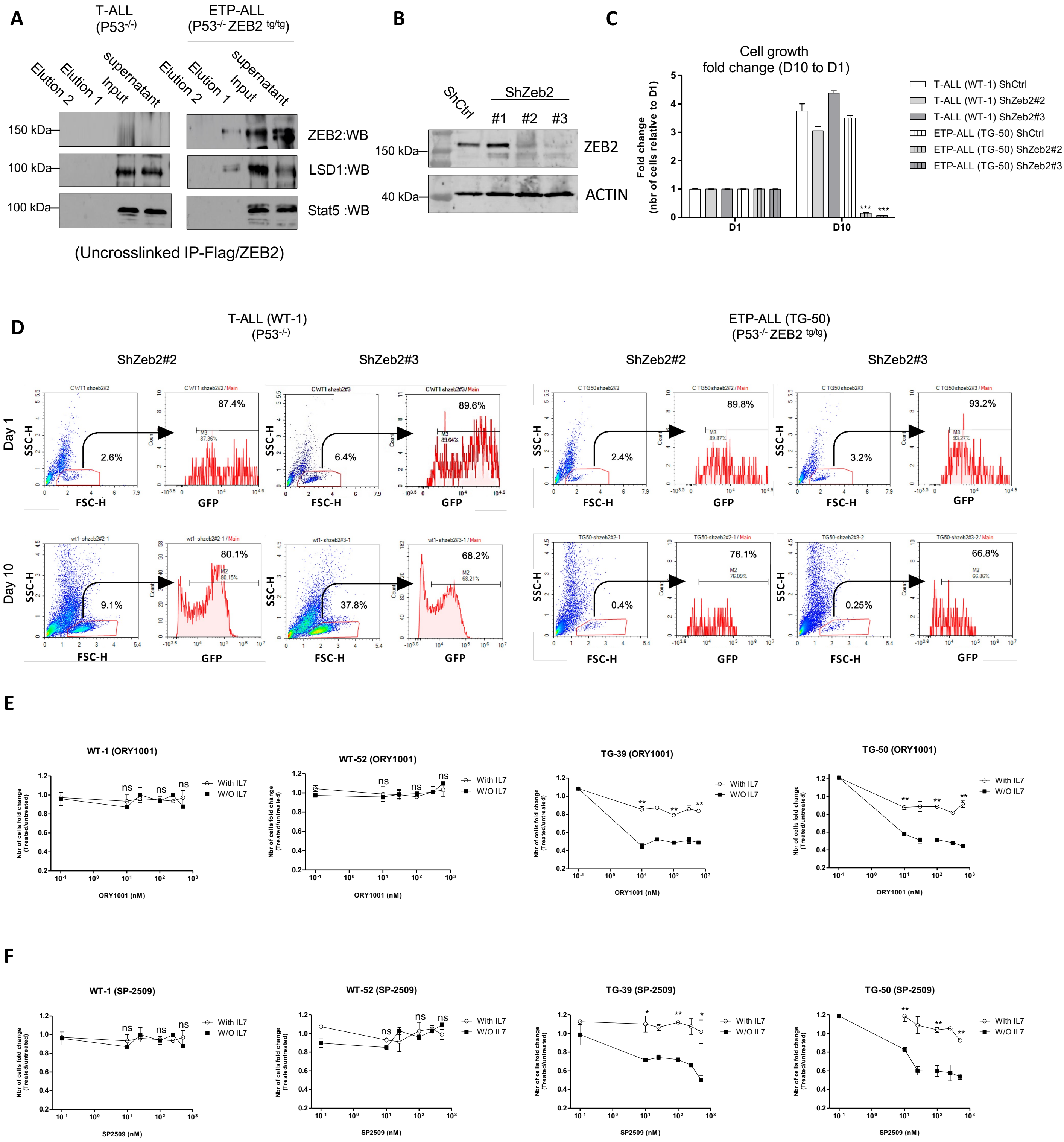
A
T-ALL
(P53-/-)
ETP-ALL
(P53-/- ZEB2 tg/tg)
Elution 2
Elution 1
Input
supernatant
150 kDa
100 kDa
100 kDa
ZEB2:WB
LSD1:WB
Stat5 :WB
(Uncrosslinked IP-Flag/ZEB2)
B
ShCtrl
ShZeb2
#1
#2
#3
150 kDa
40 kDa
ZEB2
ACTIN
C
Cell growth
fold change (D10 to D1)
Fold change
(nbr of cells relative to D1)
D1
D10
T-ALL (WT-1) ShCtrl
T-ALL (WT-1) ShZeb2#2
T-ALL (WT-1) ShZeb2#3
ETP-ALL (TG-50) ShCtrl
ETP-ALL (TG-50) ShZeb2#2
ETP-ALL (TG-50) ShZeb2#3
D
T-ALL (WT-1)
(P53-/-)
ETP-ALL (TG-50)
(P53-/- ZEB2 tg/tg)
ShZeb2#2
ShZeb2#3
Day 1
Day 10
SSC-H
FSC-H
GFP
2.6%
87.4%
6.4%
89.6%
2.4%
89.8%
3.2%
93.2%
9.1%
80.1%
37.8%
68.2%
0.4%
76.1%
0.25%
66.8%
E
WT-1 (ORY1001)
WT-52 (ORY1001)
TG-39 (ORY1001)
TG-50 (ORY1001)
Nbr of cells fold change
(Treated/untreated)
ORY1001 (nM)
With IL7
W/O IL7
F
WT-1 (SP-2509)
WT-52 (SP-2509)
TG-39 (SP-2509)
TG-50 (SP-2509)
SP2509 (nM)

Figure S2

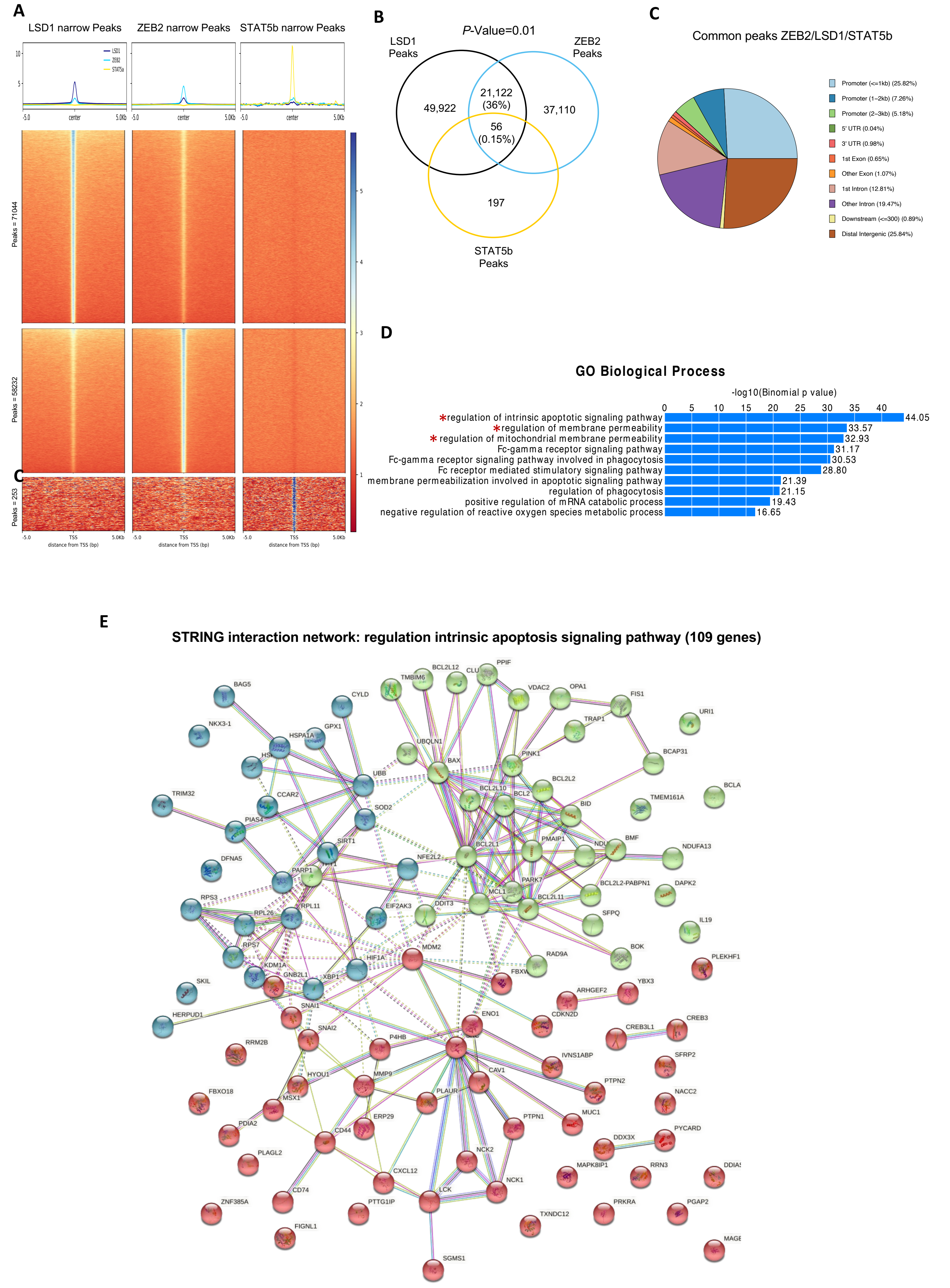
A
LSD1 narrow Peaks
ZEB2 narrow Peaks
STAT5b narrow Peaks
Peaks = 71044
Peaks = 58232
Peaks = 253
distance from TSS (bp)
B
P-Value=0.01
LSD1 Peaks
ZEB2 Peaks
STAT5b Peaks
49,922
21,122 (36%)
37,110
56 (0.15%)
197
C
Common peaks ZEB2/LSD1/STAT5b
Promoter (<=1kb) (25.82%)
Promoter (1–2kb) (7.26%)
Promoter (2–3kb) (5.18%)
5' UTR (0.04%)
3' UTR (0.98%)
1st Exon (0.65%)
Other Exon (1.07%)
1st Intron (12.81%)
Other Intron (19.47%)
Downstream (<=300) (0.89%)
Distal Intergenic (25.84%)
D
GO Biological Process
-log10(Binomial p value)
*regulation of intrinsic apoptotic signaling pathway 44.05
*regulation of membrane permeability 33.57
* regulation of mitochondrial membrane permeability 32.93
Fc-gamma receptor signaling pathway 31.17
Fc-gamma receptor signaling pathway involved in phagocytosis 30.53
Fc receptor mediated stimulatory signaling pathway 28.80
membrane permeabilization involved in apoptotic signaling pathway 21.39
regulation of phagocytosis 21.15
positive regulation of mRNA catabolic process 19.43
negative regulation of reactive oxygen species metabolic process 16.65
E
STRING interaction network: regulation intrinsic apoptosis signaling pathway (109 genes)

Figure S3

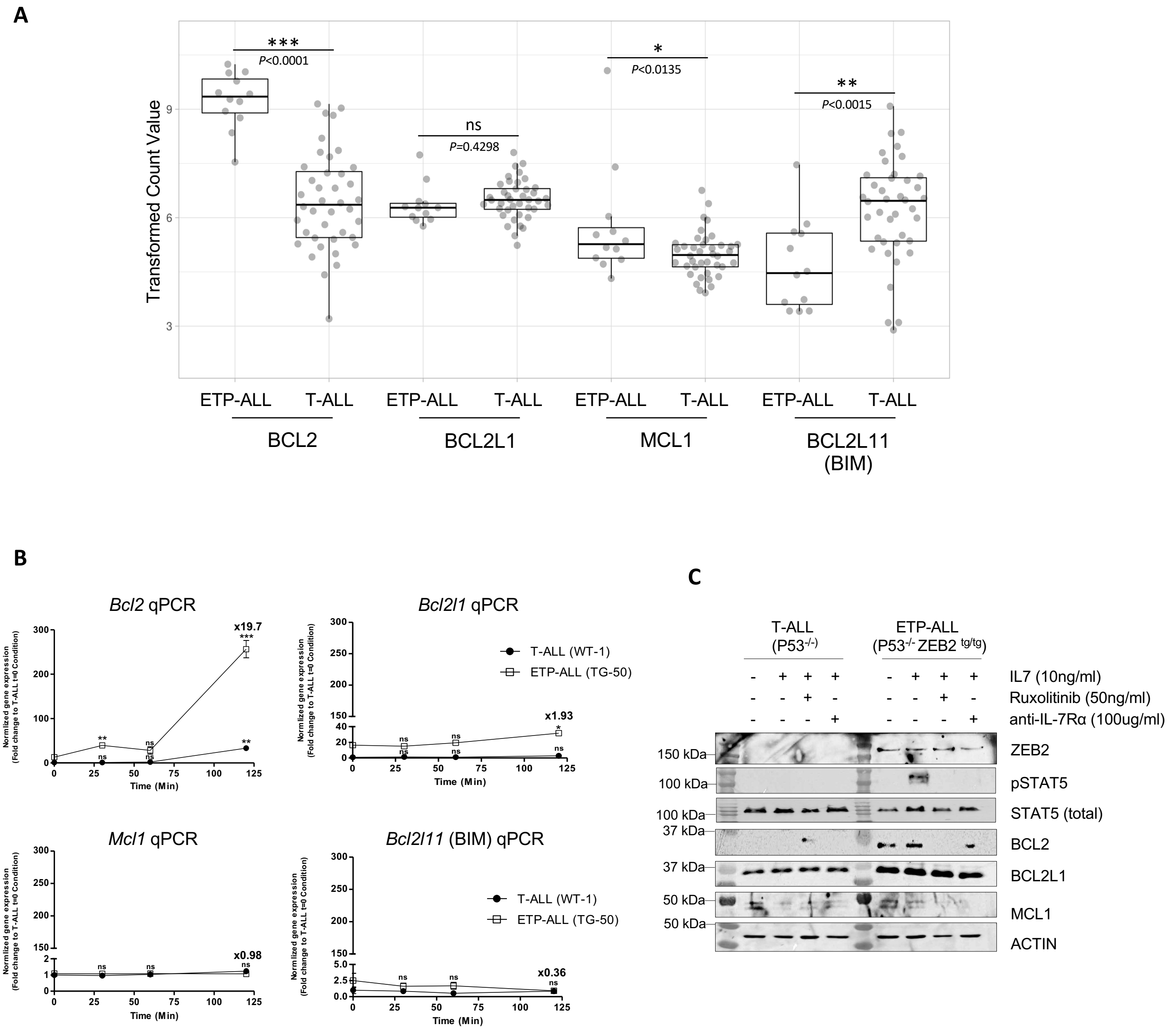
A
Transformed Count Value
***
P<0.0001
ns
P=0.4298
*
P<0.0135
**
P<0.0015
ETP-ALL
T-ALL
BCL2
BCL2L1
MCL1
BCL2L11 (BIM)
B
Bcl2 qPCR
Bcl2l1 qPCR
Mcl1 qPCR
Bcl2l11 (BIM) qPCR
Normlized gene expression (Fold change to T-ALL t=0 Condition)
Time (Min)
T-ALL (WT-1)
ETP-ALL (TG-50)
x19.7
x1.93
x0.98
x0.36
C
T-ALL (P53-/-)
ETP-ALL (P53-/- ZEB2 tg/tg)
IL7 (10ng/ml)
Ruxolitinib (50ng/ml)
anti-IL-7Rα (100ug/ml)
150 kDa
100 kDa
37 kDa
50 kDa
ZEB2
pSTAT5
STAT5 (total)
BCL2
BCL2L1
MCL1
ACTIN

Figure S4

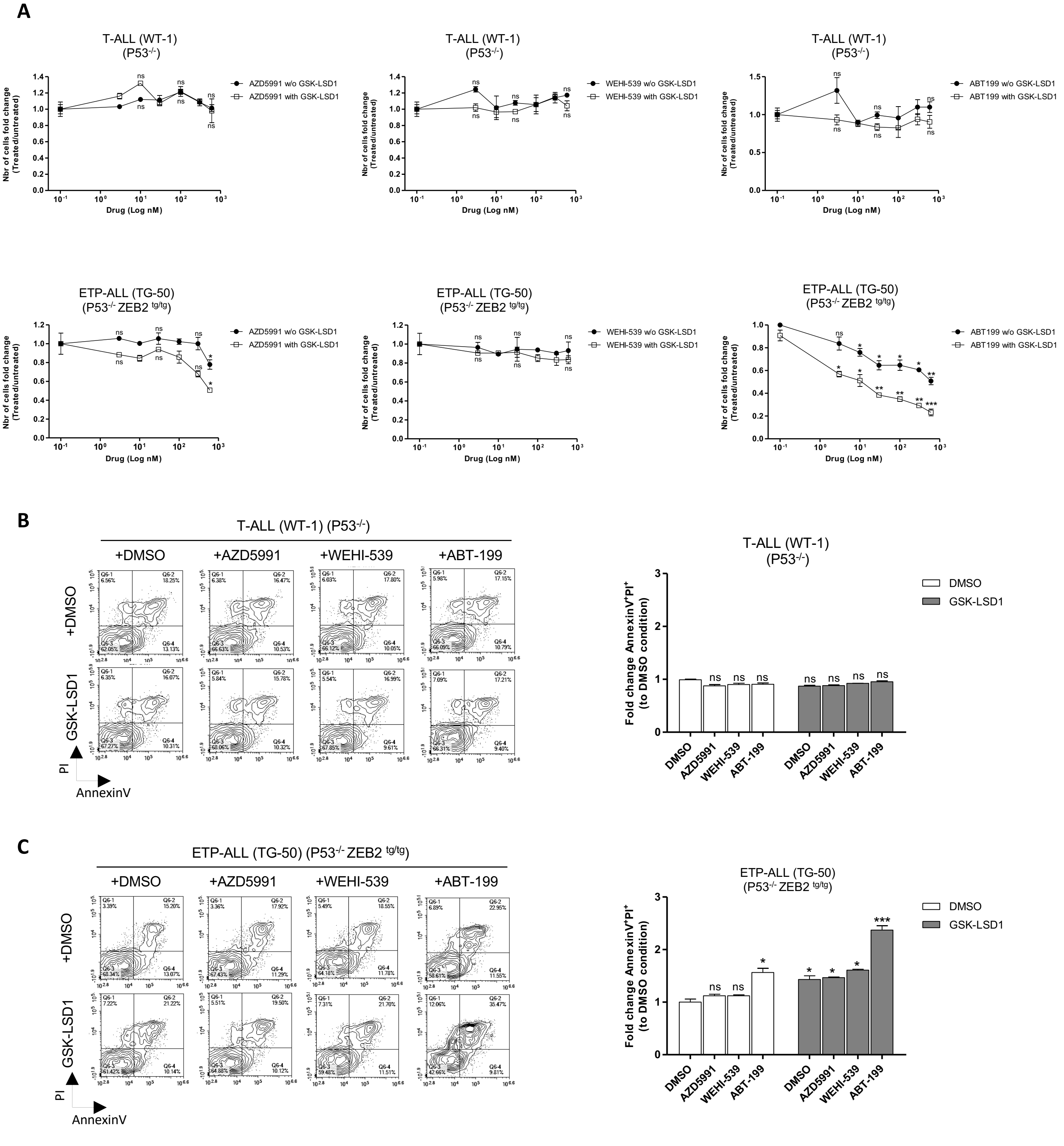

A
T-ALL (WT-1) (P53-/-)
AZD5991 w/o GSK-LSD1
AZD5991 with GSK-LSD1
WEHI-539 w/o GSK-LSD1
WEHI-539 with GSK-LSD1
ABT199 w/o GSK-LSD1
ABT199 with GSK-LSD1
Nbr of cells fold change (Treated/untreated)
Drug (Log nM)
ETP-ALL (TG-50) (P53-/- ZEB2 tg/tg)
B
T-ALL (WT-1) (P53-/-)
+DMSO
+AZD5991
+WEHI-539
+ABT-199
GSK-LSD1
PI
AnnexinV
Fold change AnnexinV+PI+ (to DMSO condition)
DMSO
GSK-LSD1
C
ETP-ALL (TG-50) (P53-/- ZEB2 tg/tg)

Figure S5

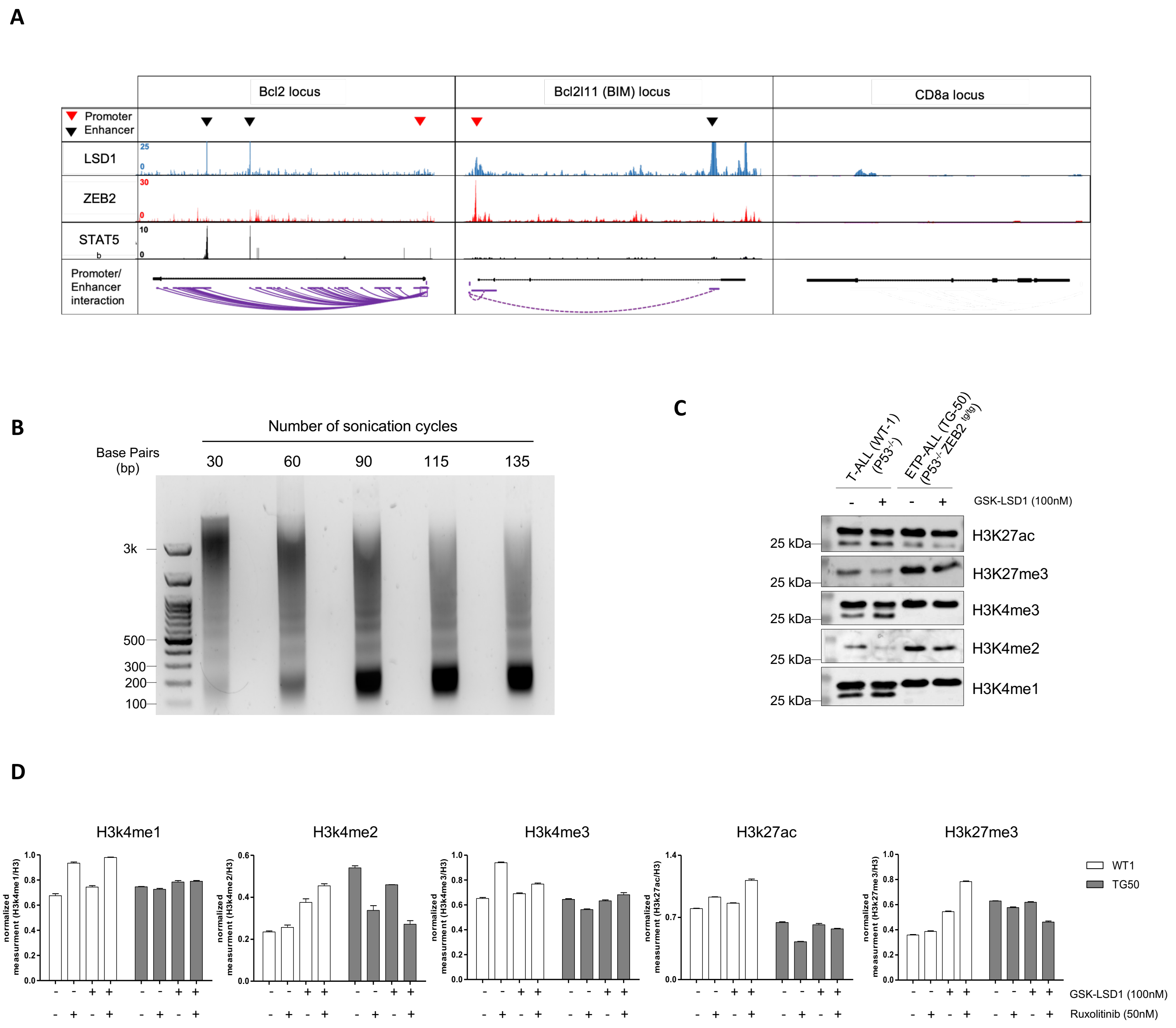
A
Bcl2 locus
Bcl2l11 (BIM) locus
CD8a locus
Promoter
Enhancer
LSD1
ZEB2
STAT5
Promoter/ Enhancer interaction
B
Number of sonication cycles
Base Pairs (bp)
30
60
90
115
135
3k
500
300
200
100
C
T-ALL (WT-1) (P53-/-)
ETP-ALL (TG-50) (P53-/-ZEB2 tg/tg)
GSK-LSD1 (100nM)
25 kDa
H3K27ac
H3K27me3
H3K4me3
H3K4me2
H3K4me1
D
H3k4me1
H3k4me2
H3k4me3
H3k27ac
H3k27me3
normalized measurment (H3k4me1/H3)
normalized measurment (H3k4me2/H3)
normalized measurment (H3k4me3/H3)
normalized measurment (H3k27ac/H3)
normalized measurment (H3k27me3/H3)
WT1
TG50
GSK-LSD1 (100nM)
Ruxolitinib (50nM)

Figure S6

A

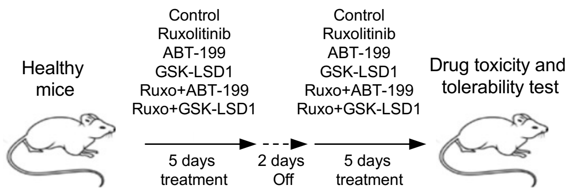

B

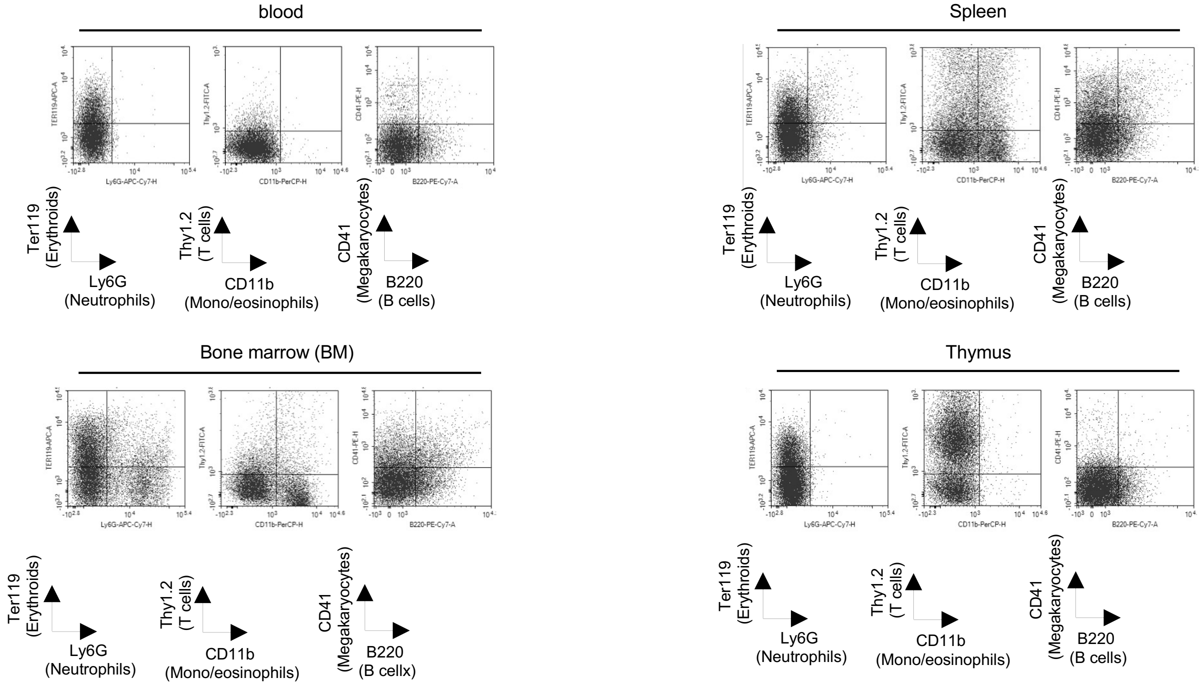

C

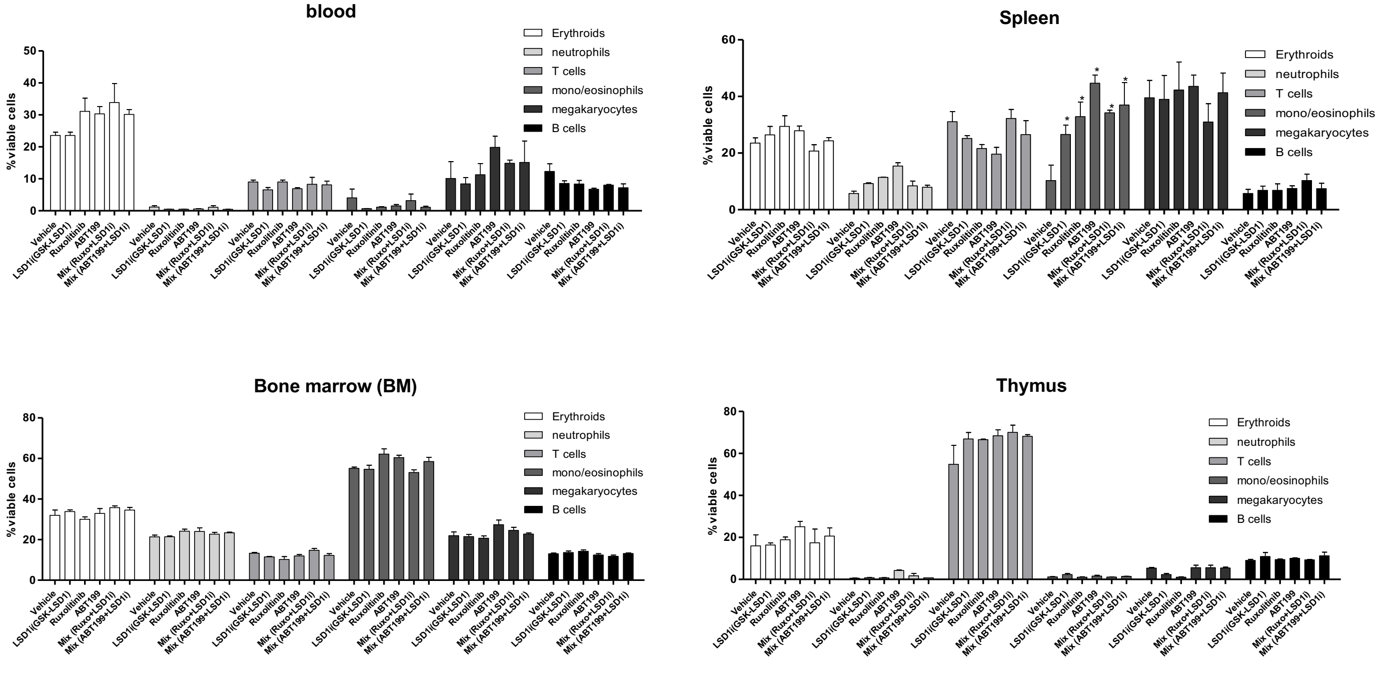

Figure S7

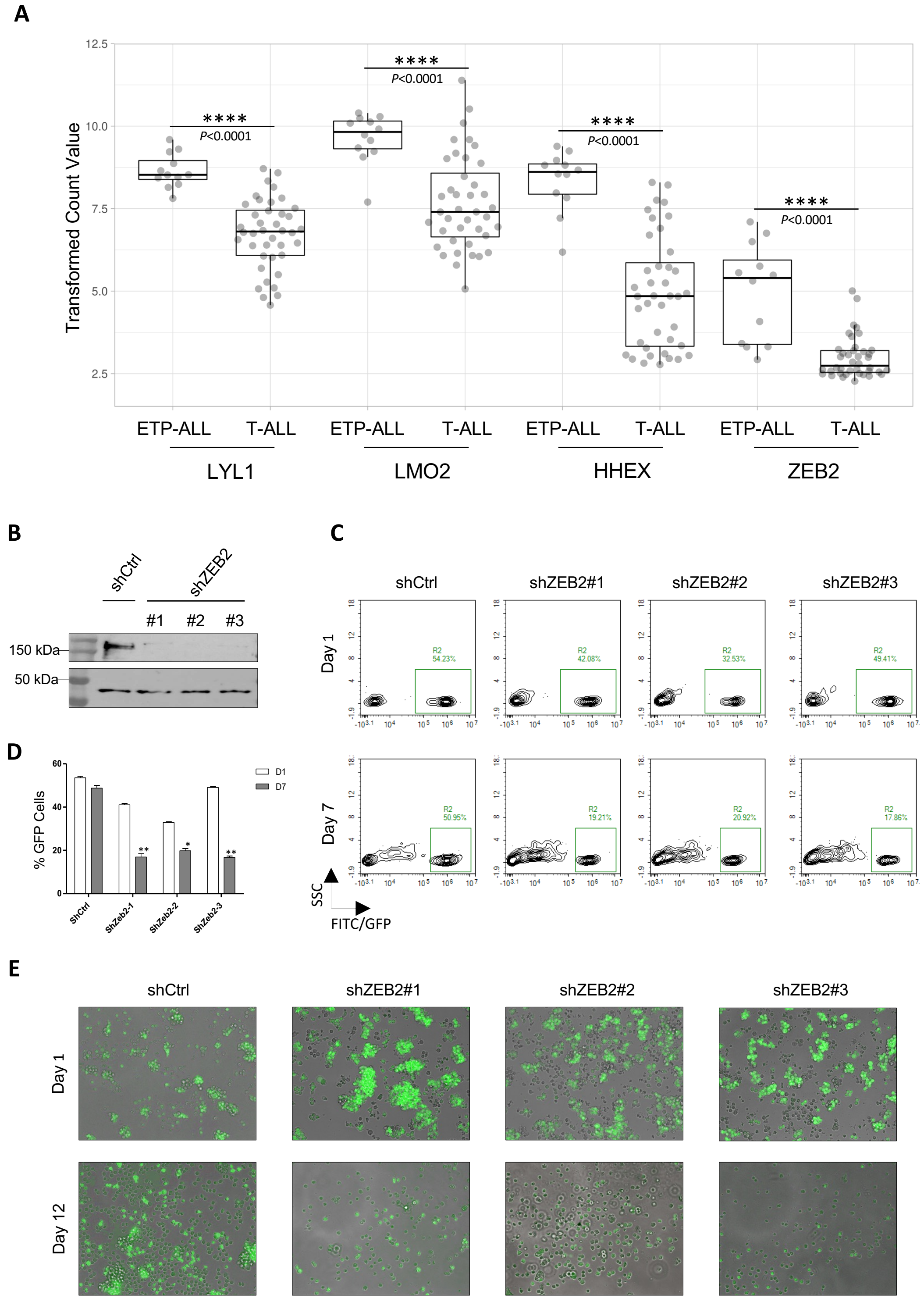
A
Transformed Count Value
12.5
10.0
7.5
5.0
2.5
****
P<0.0001
ETP-ALL
T-ALL
LYL1
LMO2
HHEX
ZEB2
B
shCtrl
shZEB2
#1
#2
#3
150 kDa
50 kDa
C
shCtrl
shZEB2#1
shZEB2#2
shZEB2#3
Day 1
Day 7
R2
54.23%
42.08%
32.53%
49.41%
50.95%
19.21%
20.92%
17.86%
SSC
FITC/GFP
D
% GFP Cells
60
40
20
0
D1
D7
shCtrl
shZeb2-1
shZeb2-2
shZeb2-3
E
shCtrl
shZEB2#1
shZEB2#2
shZEB2#3
Day 1
Day 12

Figure S8

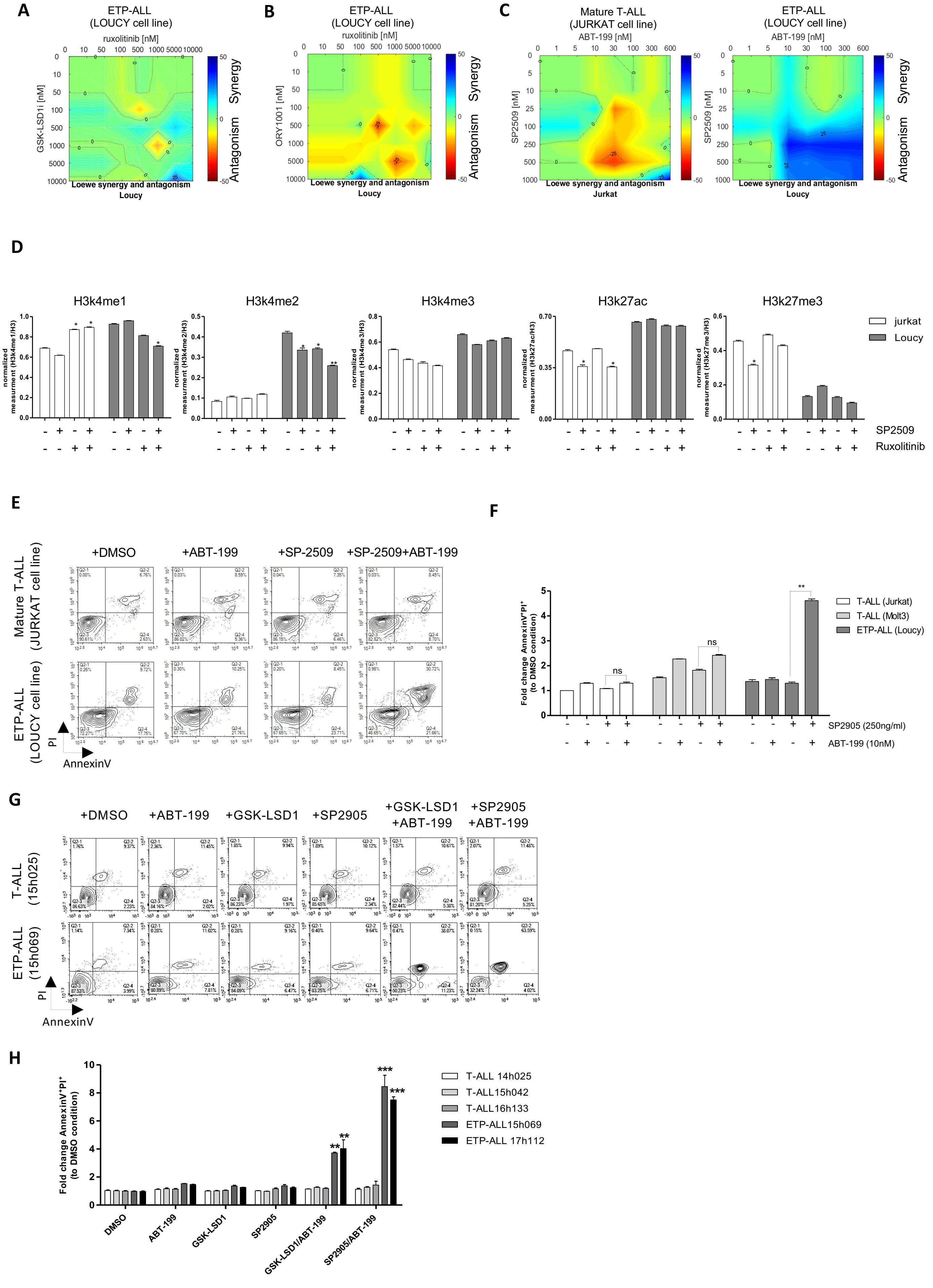
A
ETP-ALL
(LOUCY cell line)
ruxolitinib [nM]
GSK-LSD1i [nM]
Loewe synergy and antagonism
Loucy
B
ETP-ALL
(LOUCY cell line)
ruxolitinib [nM]
ORY1001 [nM]
Loewe synergy and antagonism
Loucy
C
Mature T-ALL
(JURKAT cell line)
ABT-199 [nM]
SP2509 [nM]
Loewe synergy and antagonism
Jurkat
ETP-ALL
(LOUCY cell line)
ABT-199 [nM]
SP2509 [nM]
Loewe synergy and antagonism
Loucy
Synergy
Antagonism
D
H3k4me1
H3k4me2
H3k4me3
H3k27ac
H3k27me3
jurkat
Loucy
SP2509
Ruxolitinib
E
+DMSO
+ABT-199
+SP-2509
+SP-2509+ABT-199
Mature T-ALL (JURKAT cell line)
ETP-ALL (LOUCY cell line)
PI
AnnexinV
F
Fold change AnnexinV+PI+ (to DMSO condition)
T-ALL (Jurkat)
T-ALL (Molt3)
ETP-ALL (Loucy)
SP2905 (250ng/ml)
ABT-199 (10nM)
G
+DMSO
+ABT-199
+GSK-LSD1
+SP2905
+GSK-LSD1 +ABT-199
+SP2905 +ABT-199
T-ALL (15h025)
ETP-ALL (15h069)
PI
AnnexinV
H
Fold change AnnexinV+PI+ (to DMSO condition)
T-ALL 14h025
T-ALL15h042
T-ALL16h133
ETP-ALL15h069
ETP-ALL 17h112
DMSO
ABT-199
GSK-LSD1
SP2905
GSK-LSD1/ABT-199
SP2905/ABT-199

Figure S9

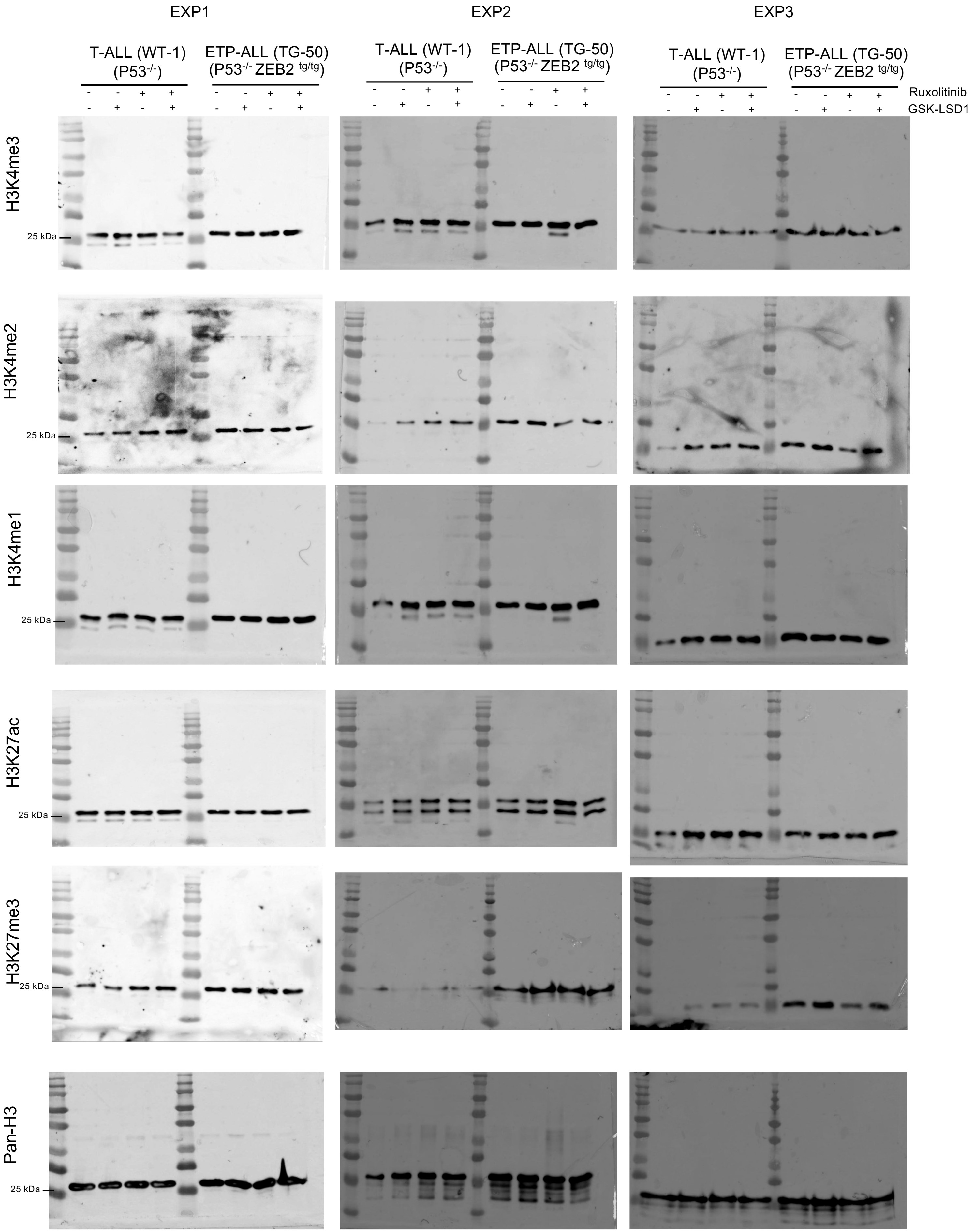
EXP1
EXP2
EXP3
T-ALL (WT-1) (P53-/-)
ETP-ALL (TG-50) (P53-/- ZEB2 tg/tg)
Ruxolitinib
GSK-LSD1
H3K4me3
H3K4me2
H3K4me1
H3K27ac
H3K27me3
Pan-H3
25 kDa

Figure S10

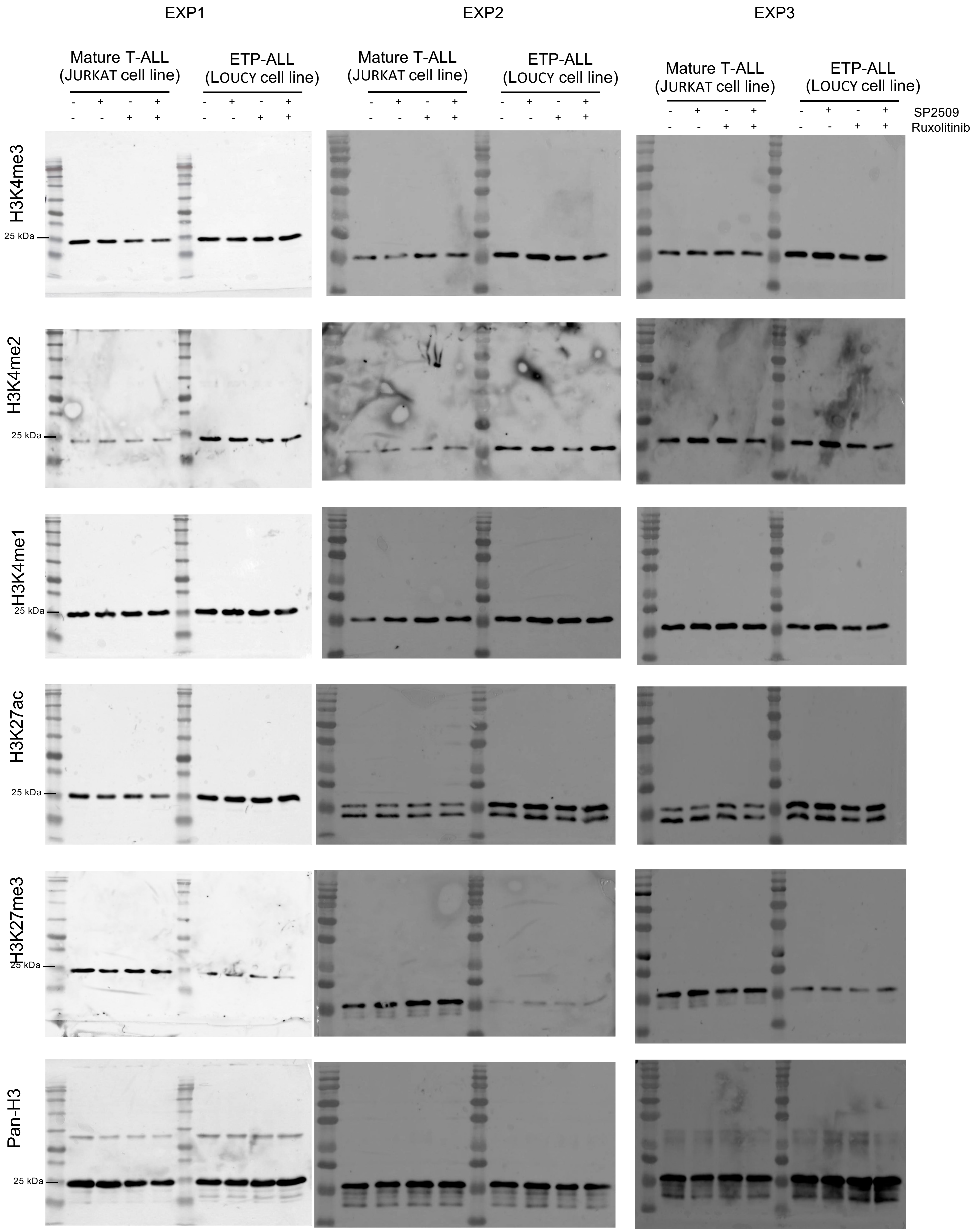

EXP1
EXP2
EXP3
Mature T-ALL (JURKAT cell line)
ETP-ALL (LOUCY cell line)
SP2509
Ruxolitinib
H3K4me3
H3K4me2
H3K4me1
H3K27ac
H3K27me3
Pan-H3
25 kDa
